# Sutureless Aortic Valve Replacement with Perceval Bioprosthesis Superior to Transcatheter Aortic Valve Implantation: A Promising Option for the Gray-Zone of Aortic Valve Replacement Procedures—A State-of-the-Art Systematic Review, Meta-Analysis, and Future Directions

**DOI:** 10.3390/jcm13164887

**Published:** 2024-08-19

**Authors:** Sadeq Ali-Hasan-Al-Saegh, Sho Takemoto, Saeed Shafiei, Senol Yavuz, Arian Arjomandi Rad, Lukman Amanov, Ali Saad Merzah, Jawad Salman, Fabio Ius, Tim Kaufeld, Bastian Schmack, Aron-Frederik Popov, Anton Sabashnikov, Arjang Ruhparwar, Alina Zubarevich, Alexander Weymann

**Affiliations:** 1 Department of Cardiothoracic, Transplantation and Vascular Surgery, Hannover Medical School, Carl-Neuberg-Straße 1, 30625 Hannover, Germanyweymann.alexander@mh-hannover.de (A.W.); 2 Center for Transplantation Sciences, Department of Surgery, Massachusetts General Hospital and Harvard Medical School, Boston, MA 02114, USA; 3 Department of Cardiovascular Surgery, Kyushu University Graduate School of Medical Sciences, Fukuoka 812-8582, Japan; 4 Department of Cardiac and Thoracic Vascular Surgery, Marburg University Hospital, 35043 Marburg, Germany; 5 Department of Cardiovascular Surgery, University of Health Sciences, Bursa Yuksek Ihtisas Training and Research Hospital, 16310 Bursa, Turkey; 6 Medical Sciences Division, University of Oxford, Oxford OX1 2JD, UK; 7 Department of Cardiothoracic Transplantation and Mechanical Circulatory Support, Royal Brompton & Harefield NHS Foundation Trust, Harefield Hospital, Harefield UB9 6JH, UK

**Keywords:** surgical aortic valve replacement, transcatheter aortic valve implantation, TAVI, sutureless aortic valve replacement, Perceval bioprothesis, sutureless, aortic valve replacement, meta-analysis

## Abstract

**Background**: The management of patients with aortic valve pathologies can sometimes fall into a “gray zone”, where the optimal treatment approach is not straightforward. The comparative benefits of sutureless aortic valve replacement (SUAVR) using the Perceval bioprosthesis versus transcatheter aortic valve implantation (TAVI) for the “gray zone” of aortic valve replacement procedures remain a topic of debate. To further explore this issue, we conducted a study with pairwise, single-arm, and Kaplan–Meier-based meta-analyses to compare the outcomes of SUAVR with the Perceval bioprosthesis versus TAVI, as well as to evaluate the efficacy, safety, and durability of SUAVR with the Perceval bioprosthesis over mid-term and long-term follow-up periods. **Methods**: The PubMed, PubMed Central, OVID Medline, Cochrane Library, Embase, and Web of Science databases were systematically searched. All study types were included, except study protocols and animal studies, without time restrictions. The final search was carried out in May 2024. **Results**: No statistically significant differences were observed in permanent pacemaker implantation (PPI) rates between the two groups. SUAVR showed a lower incidence of new-onset myocardial infarction but was associated with higher rates of new-onset atrial fibrillation and major bleeding. TAVI had higher rates of left bundle branch block and major vascular complications. **Conclusions**: Our findings show that SUAVR has a lower incidence of complications and a favorable mid-term overall survival compared to TAVI. SUAVR has more advantages compared to TAVI and can be considered a valuable and promising option for the “grey zone” of aortic valve pathologies.

## 1. Introduction

Aortic valve diseases, including aortic stenosis and regurgitation, represent significant and prevalent challenges in cardiovascular medicine. Without interventions, severe and symptomatic cases of aortic valve diseases carry a concerning prognosis, with mortality rates reaching 30–50% within the first year of follow-up [1,2]. Traditional surgical aortic valve replacement (SAVR) remains the gold standard treatment, but it carries significant risk for elderly, frail, or high-risk patients [3,4]. While transcatheter aortic valve implantation (TAVI) has emerged as a less invasive alternative therapeutic option, it is primarily indicated for elderly high-risk patients [3,5]. The management of patients with aortic valve pathologies can sometimes fall into a “gray zone”, where the optimal treatment approach is not straightforward [6,7]. These patients often have a combination of factors that make the decision between SAVR and TAVI challenging [6,7]. The “gray zone” patients may have moderate surgical risk, with comorbidities such as chronic kidney disease, chronic obstructive pulmonary disease, or previous cardiac surgeries that increase the risk of conventional surgery but do not necessarily make them high-risk enough for TAVI. Additionally, they may have anatomical considerations that make the procedural approach more complex, such as a bicuspid aortic valve, severe aortic root disease, or extensive calcification of the aortic annulus [6,7]. 

The Perceval bioprosthesis is a unique, self-anchoring aortic valve replacement device that can be implanted through a minimally invasive approach without extensive surgical suturing [8,9]. This innovative design aims to combine the benefits of reduced procedural complexity and risk with the durability and hemodynamic performance of a bioprosthetic valve replacement [8,9]. In the context of the “gray zone” of aortic valve replacement procedures, sutureless aortic valve replacement (SUAVR) with the Perceval bioprosthesis could be an alternative promising approach, potentially offering a middle ground between the traditional surgical and transcatheter techniques. 

The latest report from the Society of Thoracic Surgeons Adult Cardiac Surgery Database demonstrated that the need for surgical strategies, including post-TAVI redo SAVR, is rapidly increasing [10]. The risk of transcatheter aortic valve explant has been reported to be higher than predicted [10]. Outcomes will be worse in the long term if TAVI is broadly applied to the “gray zone” of aortic valve pathologies at lowering age and risk profiles in the absence of longitudinal evidence [10].

The comparative benefits of SUAVR using the Perceval bioprosthesis versus TAVI for the “gray zone” of aortic valve replacement procedures remain a topic of debate. To further explore this issue, we conducted a study with pairwise, single-arm, and Kaplan–Meier-based meta-analyses of published studies to compare the outcomes of SUAVR with the Perceval bioprosthesis versus TAVI, as well as to evaluate the efficacy, safety, and durability of SUAVR with the Perceval bioprosthesis over mid-term and long-term follow-up periods.

## 2. Materials and Methods

- Eligibility criteria

This study, including pairwise and single-arm meta-analyses, was conducted in accordance with the Cochrane Collaboration published guidelines and the Preferred Reporting Items for Systematic Reviews and Meta-Analyses (PRISMA) guidelines [11]. For the first part of this study, a meta-analysis comparing SUAVR and TAVI was conducted. The first part of this study was focused on providing reliable comparisons of outcomes and complications between the SUAVR and TAVI procedures in the early and mid-term follow-up periods. In addition, subgroup analyses were performed in relation to minimally invasive and risk profiles of the patients to investigate the comparison between TAVI and SUAVR for the “gray zone” of aortic valve replacement procedures. The patients belong to a “gray zone”, which includes cases where they are at high risk for SAVR but where they are not really inoperable.

Therefore, the study question was formulated using the Population, Intervention, Comparison, Outcome, and Study (PICOS) design strategy. Studies were included in the analysis if they met the following criteria:

- Population: all patients suffered from aortic valve pathologies;
- Intervention: SUAVR with Perceval bioprosthesis;
- Comparator: TAVI;
- Outcome: early and mid-term outcomes and complications after both procedures;
- Study design: Original articles were included in the initial assessment. Experimental studies, case reports, conference summaries, letters, editorials, reviews, and general overviews were excluded.

For the second part of this study on the efficacy, safety, and durability of SUAVR with the Perceval bioprosthesis, single-arm and Kaplan–Meier-based meta-analyses of included studies with mid-term and long-term reports were performed to assess more precisely the selection of SUAVR as a promising option for the “gray zone” of aortic valve replacement procedures. Studies were included in which patients had undergone SUAVR with a Perceval bioprosthesis, either alone or with a concomitant procedure. The studies could be randomized control trials or observational studies. They had to report results for up to five years, with complete survival data required. Studies were excluded if they included valves other than the Perceval valve or if the time to event was insufficient (defined as five years). If the reports on SUAVR are reported for up to 5 years, they are considered as follow-up for mid-term outcomes, and longer than five years can be regarded as long-term outcomes. To ensure the integrity of the meta-analysis, we carefully screened the included studies to identify and remove any duplicate publications or overlapping patient data. This was an important step to avoid the risk of analyzing the same individuals more than once, which could skew the results. When we encountered instances where multiple reports appeared to be drawn from the same patient population, we included only the most recent publication to prevent over-representing those cases. By taking these measures, we aimed to provide a robust synthesis of the available evidence that accurately reflects the underlying outcomes.

- Literature search

PubMed, PubMed Central, OVID Medline, Cochrane Library, Embase, and Web of Science databases were systematically searched using a combination of the following search terms: “Perceval” OR “Sutureless” OR “Aortic Valve bioprosthesis” OR “Perceval bioprosthesis” OR “transcatheter” OR “transfemoral” OR “transapical” OR “trans-subclavian” OR “conventional” OR “standard” OR “minimally invasive” AND “aortic valve” OR “aortic-valve” OR “aortic valve stenosis” OR “aortic valve pathology” AND “implantation” OR “replacement” OR “procedure” OR “treatment”. No restrictions were imposed on the publication year when the literature search was conducted. The final search was carried out in May 2024. Additionally, the reference lists of the retrieved articles were carefully reviewed to identify any other relevant studies that may have been missed in the initial search.

- Data extraction

Based on the predefined PICOS criteria, all titles and abstracts were independently screened by two authors (SAHS, LA, ASM). Eligible full-text articles were then independently reviewed by four authors (SS, SY, ST, and SAHS) to select articles for inclusion and extract the data. Any disagreement was resolved by the senior authors (AW, AZ).

- Outcome measures

To facilitate the comparison between SUAVR and TAVI in the first part of the study, we extracted the following data from the included articles: study design, country of origin, sample size, median follow-up, demographic data, type of aortic valve pathologies, risk profiles of patients, type of surgery, valve prosthesis sizes, duration of cardiopulmonary bypass (CPB), time of aortic cross-clamping (ACC), TAVI access, type of valves for TAVI procedure, mean valve gradient (MVG), and peak valve gradient (PVG). The surgical access for the implantation of sutureless bioprostheses in the included studies was either a full sternotomy or minimally invasive, via ministernotomy or minithoracotomy. In addition, details of complications after SUAVR and TAVI were recorded, such as any type of paravalvular leak (PVL), severe PVL, permanent pacemaker implantation (PPI), prosthesis–patient mismatch (PPM), the occurrence of new-onset atrial fibrillation (NOAF), the occurrence of coronary obstruction, left bundle branch block (LBBB), the occurrence of stroke, acute kidney injury (AKI), the need for dialysis, new-onset myocardial infarction (NOMI), major hemorrhage, rate of conversion to standard conventional surgical valve replacement, rate of annulus rupture, recurrence of vascular complications, length of stay in intensive care unit (ICU) and hospital, post-operative aortic valve area, and device success. In-hospital mortality, 30-day mortality, and overall survival were also recorded. For the first part of our study, we attempted to compare the above outcomes, complications, and mortality rates in the early and mid-term periods.

For the second part of this study, the following additional data were extracted: MVG, PVG, and survival rates in each year from 1 to 10 years after SUAVR (if possible, when data were available). We also collected information on midterm and long-term complications such as PVL, severe PVL, structural valve deterioration (SVD), infective endocarditis, the need for the explantation of the Perceval bioprosthesis in general, the need for explantation due to SVD, PVL or endocarditis, and the occurrence of strokes.

The data on various outcomes, complications, mortality, and survivorship rates were categorized based on the duration of follow-up. The early outcomes were defined as data reported within a hospital stay of up to 30 days. The mid-term outcomes were data reported over a period of 1 to 5 years. Finally, the long-term outcomes were data reported more than 5 years after the intervention.

- Ethics

Since our study is based on previously published literature and did not involve any interaction with human subjects, there were no medical ethics issues that needed to be addressed.

- Statistical analysis

Review Manager software (RevMan version 5.3.5; The Cochrane Collaboration, The Nordic Cochrane Centre, Copenhagen, Denmark) was used to perform meta-analyses. Because of expected clinical heterogeneity between the included studies, the Mantel–Haenszel random-effects model was used. Dichotomous data are presented as odd ratios (ORs) and continuous data as weighted mean differences (MDs). Summary effect measures are presented with corresponding 95% confidence intervals (CI). The methods of Hozo et al. were applied to estimate mean and standard deviation values for studies that reported only medians and ranges. For the single-arm meta-analysis, analyses of proportions were conducted for data using a random effects model to calculate pooled incidences of complications and survival rates and their confidence intervals (CI) using per protocol and intention to treat data when available. Statistical heterogeneity was evaluated using the I^2^ statistic. An I^2^ value between 0% and 25% indicates insignificant heterogeneity, 26% and 50% low heterogeneity, 51% and 75% moderate heterogeneity, and 76% and 100% high heterogeneity. A fixed-effects model was used when the I^2^ was < 50%, and a random-effects model was used when it was >50%.

## 3. Results

**Figure 1:**
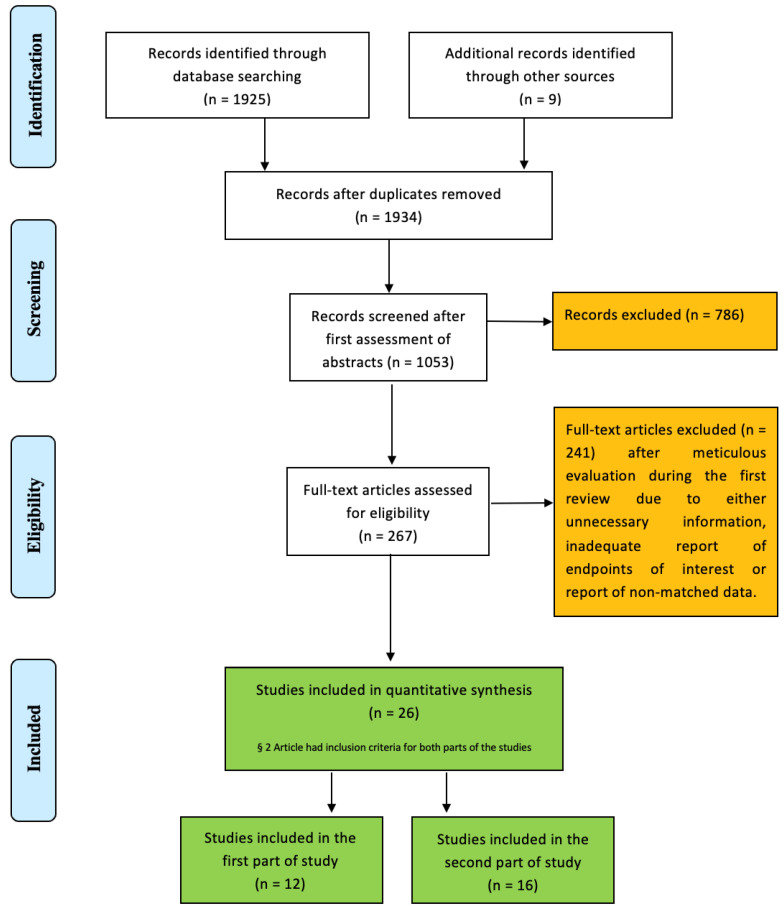
PRISMA flow chart of study selection.

The initial literature search identified a total of 1934 articles after removing any duplicates (Figure 1). After a full-text evaluation of the remaining 267 articles for both parts of the study, 241 were excluded from further analysis because the studies were non-comparative, did not include information about the Perceval bioprosthesis, included irrelevant or redundant information, or did not adequately report the primary outcomes of interest, especially the mid- and long-term outcomes. Finally, 12 studies [12,13,14,15,16,17,18,19,20,21,22,23] with 3764 patients were included in the comparative pairwise meta-analysis comparing TAVI and SUAVR in the first part (Table 1 and Supplementary Tables S1–S7), and 16 studies [17,20,24,25,26,27,28,29,30,31,32,33,34,35,36,37] with 7254 patients were included in the single-arm binary meta-analysis in the second part of this study (Table 2 and Supplementary Tables S8–S11).

- First Part of the Study

*Comparison of Early Outcomes between SUAVR and TAVI*

*1. Early Paravalvular Leak (PVL)*

**Figure 2:**
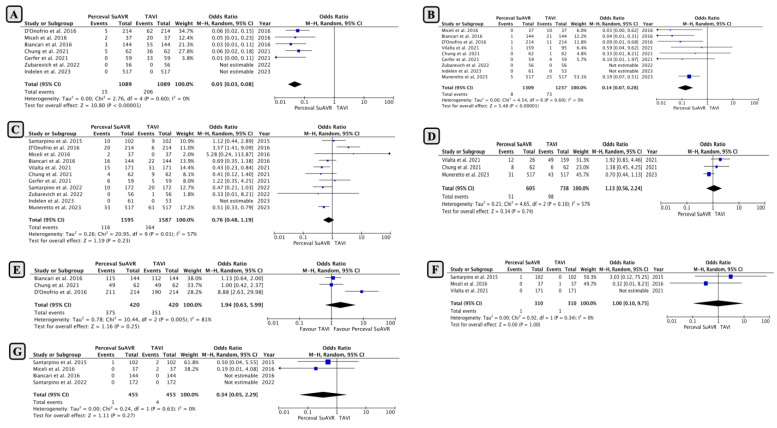
Comparison of early outcomes between SUAVR and TAVI ((**A**) mild paravalvular leak; (**B**) moderate to severe paravalvular leak; (**C**) pacemaker implantations; (**D**) prosthesis-patient mismatch; (**E**) device success rate; (**F**) annulus rupture; (**G**) conversion to a conventional surgical procedure).

Early mild PVL occurred in 1.37% (15/1089 patients) in the SUAVR group, which was significantly lower than the 18.9% (206/1089 patients) in the TAVI group. Pooled analysis revealed that SUAVR had a significantly lower rate of mild PVL, with an OR of 0.05 (95% CI: 0.03−0.08; *p* < 0.00001; Figure 2A) using a random-effects model (I2 = 0%, Chi2 = 2.67; *p* = 0.6).

Early moderate to severe PVL was reported in nine studies with a total of 2546 patients. The incidence of moderate to severe PVL was 0.6% in patients who underwent SUAVR compared to 5.9% in those who underwent TAVI (Figure 2B; OR: 0.14; 95% CI: 0.07−0.28; *p* < 0.00001), with minimal heterogeneity between studies (I2 = 0%; *p* = 0.6).

*2. Pacemaker Implantation and Prosthesis–Patient Mismatch (PPM)*

A permanent pacemaker was implanted in 7.27% (116 out of 1595) of patients in the SUAVR group compared to 10.3% (164 out of 1587) of patients who underwent TAVI procedures. The pooled analysis showed an increasing trend towards more pacemaker implantations in the TAVI group, but this difference was not statistically significant (OR 0.76; 95% CI: 0.48−1.19; *p* = 0.23; Figure 2C). However, there was considerable heterogeneity between the studies (I2 = 57%; *p* = 0.23).

The incidence of prosthesis–patient mismatch (PPM) was 8.4% (51 of 605 cases) in the SUAVR group, compared to 13.2% (98 of 738 cases) in the TAVI group. However, the difference between the groups was not statistically significant (OR: 1.13; 95% CI: 0.56−2.24; *p* = 0.74; Figure 2D).

*3. Device Success and Conversion to Conventional Surgery*

The success rate of the devices was statistically similar for both procedures, although SUAVR tended to be more successful (89.2% in SUAVR and 83% in TAVI with OR: 1.94; 95% CI: 0.63−5.99; *p* = 0.25; Figure 2E).

No difference was observed between SUAVR and TAVI in the context of the occurrence of an annulus rupture (Figure 2F). According to the pooled analysis, TAVI was associated with a higher rate of conversion to a conventional surgical procedure compared to SUAVR, but this difference was not statistically significant (0.21% in SUAVR and 0.87% in TAVI with OR: 0.34; 95% CI: 0.05−2.29; *p* = 0.27; Figure 2G).

*4. New onset myocardial infarction (NOMI) and New-Onset Atrial Fibrillation (NOAF)*

**Figure 3:**
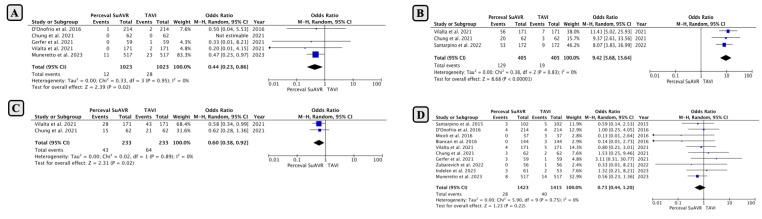
Comparison of early outcomes between SUAVR and TAVI (continued) ((**A**) New onset myocardial infarction; (**B**) New-Onset Atrial Fibrillation; (**C**) left bundle branch block; (**D**) Stroke).

The incidence of NOMI occurred in 1.17% of patients following SUAVR and 2.73% of patients following TAVI. The pooled analysis employing a random-effects model indicated that SUAVR had a significantly lower incidence of NOMI compared to TAVI (OR: 0.44, 95% CI: 0.23−0.86; *p* < 0.01; Figure 3A), without considerable heterogeneity (I2 = 0%, Chi2 = 0.33; *p* = 0.95).

The occurrence of NOAF was significantly higher in the SUAVR group compared to the TAVI group (31.8% vs. 4.6%, respectively, with OR: 9.42; 95% CI: 5.68−15.64; *p* < 0.00001; Figure 3B).

*5. Left Bundle Branch Block (LBBB) and Stroke*

TAVI was associated with a higher risk of the occurrence of an LBBB in comparison to SUAVR (18.4% in SUAVR and 27.4% in TAVI with OR: 0.60; 95% CI: 0.38−0.92; *p* = 0.02; Figure 3C).

Ten studies reported the incidence of stroke following TAVI or SUAVR, with a total of 2838 included patients. The pooled analysis showed an increased risk of stroke in the TAVI group compared to the SUAVR group (1.96% in SUAVR and 2.82% in TAVI with OR: 0.73; 95% CI: 0.44−1.20; *p* = 0.22; Figure 3D). However, this difference was not statistically significant. The heterogeneity between the studies was not considerable (I2 = 0%, Chi2 = 5.90; *p* = 0.75).

*6. Major Bleeding and Vascular Complications*

**Figure 4:**
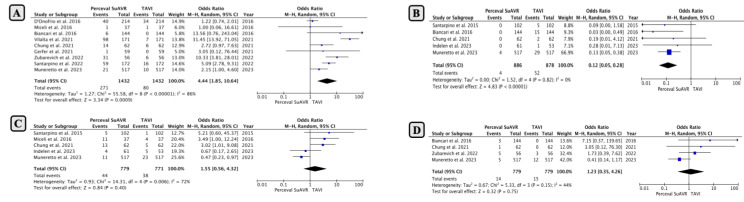
Comparison of early outcomes between SUAVR and TAVI (continued) ((**A**) Major bleeding; (**B**) Major vascular complications; (**C**) Acute Kidney Injury; (**D**) Hemodialysis).

An early rate of major bleeding was reported in nine studies with a total of 2864 included patients. The pooled analysis showed that the rate of major bleeding was significantly lower in patients who underwent TAVI compared to SUAVR as the surgical option (OR: 4.44; 95% CI: 1.85−10.64; *p* = 0.0009; Figure 4A).

The pooled analysis also indicated a significantly higher risk of major vascular complications in patients who underwent TAVI compared to SUAVR (0.45% in SUAVR and 5.9% in TAVI with OR: 0.12; 95% CI: 0.05−0.28; *p* < 0.00001; Figure 4B).

*7. Acute Kidney Injury (AKI) and Hemodialysis*

No difference was observed between SUAVR and TAVI in the context of the incidence of AKI and the need for hemodialysis (Figure 4C,D).

*8. Length of Stay and Early Echocardiographic Findings*

**Figure 5:**
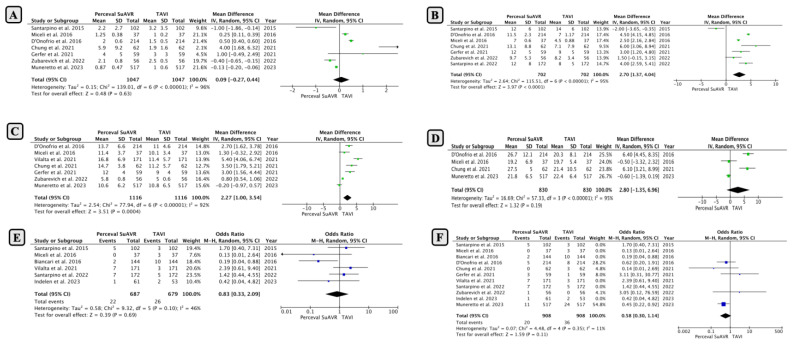
Comparison of early outcomes between SUAVR and TAVI (continued) ((**A**) ICU stay; (**B**) hospital stays; (**C**) Mean valve gradient; (**D**) Peak valve gradient; (**E**) In-hospital mortality; (**F**) 30-day mortality).

The pooled analysis employing a random effects model indicated that TAVI had no advantages over SUAVR in the context of the length of ICU stay (MD: 0.09, 95% CI: −0.27 to 0.44; *p* = 0.63; Figure 5A). On the other side of the coin, SUAVR as a surgical technique had significantly longer hospital stays compared to TAVI (MD: 2.70, 95% CI: 1.37 to 4.04; *p* < 0.00001; Figure 5B).

In the context of early echocardiographic findings, our analyses indicated that SUAVR had a higher early MVG with an MD of 2.27 (95% CI: 1.0 to 3.54; *p* = 0.0004; Figure 5C) and a non-significantly higher early PVG with an MD of 2.80 (95% CI: −1.35 to 6.96; *p* = 0.19; Figure 5D) compared to TAVI, respectively (Supplementary Table S4).

*9. Mortality Rates*

Although in-hospital mortality was not different between SUAVR and TAVI (3.2% in SUAVR and 3.8% in TAVI with OR: 0.83; 95% CI: 0.33−2.09; *p* = 0.69; Figure 5E), there was a trend to more risk of 30-day mortality in TAVI compared to SUAVR (2.2% in SUAVR and 3.96% in TAVI with OR: 0.58; 95% CI: 0.30−1.14; *p* = 0.11; Figure 5F).

*Subgroup Analysis Based on Minimally Invasive SUAVR*

The subgroup analysis revealed some differences from the overall analysis of early outcomes. First, for minimally invasive SUAVR (MI-SUAVR), the length of hospital stay was not significantly different from TAVI (MD: 1.57, 95% CI: −1.04 to 4.18; *p* = 0.24; Supplemental Figure S1A). This contrasts with the overall analysis, which found that SUAVR had significantly longer hospital stays compared to TAVI. Second, the subgroup analysis showed no significant difference in early MVG between MI-SUAVR and TAVI (MD: 0.37, 95% CI: −1.05 to 1.80; *p* = 0.61; Supplemental Figure S1B). This differed from the overall analysis, which found that SUAVR had a higher early MVG compared to TAVI.

*Subgroup Analysis Based on Intermediate-High-Risk Clinical Profiles*

However, after removing a study that reported on the clinical outcomes of TAVI in low-risk patients with aortic pathologies, the analyses were repeated, and there was no dramatic difference in outcomes compared to the overall analysis of early outcomes.

*Comparison of Mid-Term Outcomes Between SUAVR and TAVI*

Regarding PPI, the rates were 12.9% in the SUAVR group and 18.4% in the TAVI group. The pooled analysis showed an increasing trend towards more pacemaker implantations in the TAVI group at mid-term follow-up (OR 0.66; 95% CI: 0.39−1.09; *p* = 0.1; Supplemental Figure S2A). Additionally, the pooled analysis found no significant difference in the occurrence of stroke between SUAVR and TAVI at mid-term follow-up (OR: 1.63; 95% CI: 0.72−3.69; *p* = 0.24; Supplemental Figure S2B). Importantly, the overall survival rate at the mid-term follow-up was significantly better for SUAVR compared to TAVI (85.2% vs. 78.9%) with an OR of 1.89 (95% CI: 0.98−3.65; *p* = 0.06; Supplemental Figure S2C). This suggests that SUAVR may confer a survival advantage over TAVI in the mid-term.

- Second Part of the Study

*Mid-term Outcomes Following SUAVR*

*1. Paravalvular Leak (PVL)*

**Figure 6:**
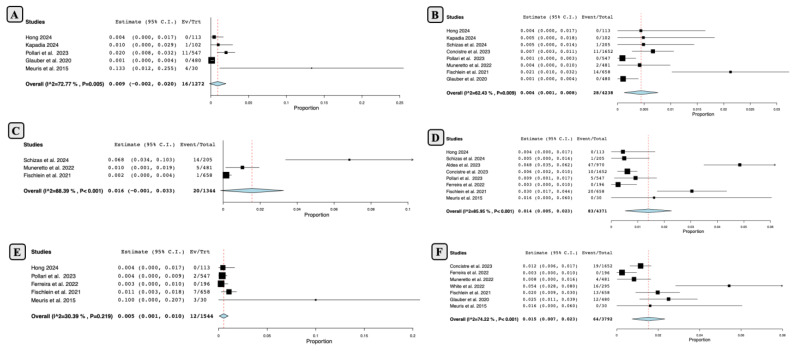
Mid-term outcomes following SUAVR ((**A**) Paravalvular leak of any type; (**B**) Severe Paravalvular leak of any type; (**C**) Permanent Pacemaker Implantation; (**D**) Structural Valve Deterioration; (**E**) Endocarditis; (**F**) Stroke).

The overall incidence of PVL of any type in patients who underwent SUAVR was relatively low, at around 1.25% (16 out of 1272 SUAVR cases) with an estimated rate of 0.9% (95% CI: − 0.2% to 2.0%, SE of 0.006, and *p* = 0.10; Figure 6A). There was considerable heterogeneity among the studies (*p* = 0.005; I2 = 72.7%).

The rate of severe PVL was even lower, at 0.6% (28 out of 4238 SUAVR cases) at mid-term follow-up. The estimated severe PVL rate was 0.4% (95% CI: 0.1% to 0.8%, SE of 0.002, and *p* = 0.007; Figure 6B).

*2. Permanent Pacemaker Implantation (PPI)*

The pooled analysis showed that the rate of new PPI at the mid-term follow-up was 1.48% (20 out of 1344) of SUAVR patients. The single-arm meta-analysis estimated the rate of PPI with SUAVR to be 1.6% (95% CI: −0.1% to 3.3%, SE of 0.009, and *p* = 0.07; Figure 6C).

*3. Structural Valve Deterioration (SVD)*

According to the pooled data, SVD occurred in 1.89% (83 of 4371 SUAVR cases) during the mid-term follow-up period. The one-arm meta-analysis estimated the SVD rate in SUAVR to be 1.4% (95% CI: 0.5% to 2.3%, SE of 0.004, and *p* = 0.002; Figure 6D).

*4. Endocarditis*

Endocarditis occurred between 1 and 5 years in 12 of 1544 implanted Perceval bioprostheses (0.77%) with a rate of 0.5% (95% CI: 0.1% to 1.0%, SE of 0.002, and *p* = 0.027; Figure 6E).

*5. Stroke*

During the mid-term follow-up period, 1.6% (64 of 3792) of patients who underwent SUAVR suffered a stroke. The single-arm meta-analysis estimated the stroke rate for SUAVR to be 1.5% (95% CI: 0.7% to 2.3%, SE of 0.004, and *p* = 0.001; Figure 6F).

*6. Explantation of Perceval Bioprosthesis.*

The need for explantation of the Perceval bioprosthesis between 1 and 5 years after SUAVR was generally 1.64% (74 of 4486 cases with an estimated rate of 1.4% and 95% CI: 0.6% to 2.1%, SE of 0.004, and *p* = 0.001; Supplementary Figure S3A).

Explantation due to PVL was 0.4% (11 of 2662 cases with an estimated rate of 0.3% and 95% CI: 0.1% to 0.5%, SE of 0.001, and *p* = 0.003; Supplementary Figure S3B).

Explantation due to endocarditis was 0.37% (3 of 805 cases with an estimated rate of 0.3% and 95% CI: 0.0% to 0.7%, SE of 0.002, and *p* = 0.108; Supplementary Figure S3C).

Explantation due to SVD was 1.4% (45 of 3152 cases with an estimated rate of 1.1% and 95% CI: 0.2% to 2.0%; Supplementary Figure S3D).

*Long-term Outcomes Following SUAVR*

*1. Paravalvular Leak (PVL)*

In our single-arm analysis of 1799 patients who underwent SUAVR, 11 cases (0.61%) experienced new-onset severe PVL during the long-term follow-up period with an estimated rate of 0.5% (95% CI of 0.0% to 1.0%, SE of 0.003, and *p* = 0.107; Supplementary Figure S4A).

*2. Permanent Pacemaker Implantation (PPI)*

Two studies reported on PPI between 5 and 10 years and showed that long-term PPI was required in 14 of 648 cases (2.1%) after SUAVR (estimated rate of 2.6% and 95% CI: 0.0% to 5.6%, SE of 0.015, and *p* = 0.082; Supplementary Figure S4B).

*3. Structural Valve Deterioration (SVD)*

Long-term SVD occurred in 2.72% (49 of 1799 cases) of patients who underwent SUAVR, with an estimated rate of 2.7% (95% CI of 1.6% to 3.8%, SE of 0.005, and *p* = 0.001; Supplementary Figure S4C).

*4. Stroke*

During the long-term follow-up, 26 of 1432 (1.81%) SUAVR patients suffered from an episode of stroke. The single-arm meta-analysis estimated the long-term stroke rate for SUAVR to be 1.7% (95% CI: 0.1% to 3.3%, SE of 0.008, and *p* = 0.032; Supplementary Figure S4D).

*5. Endocarditis*

The risk of endocarditis in the long-term course was 1.67% (24 of 1432 cases with an estimated rate of 1.6% and 95% CI: 1.0% to 2.3%, SE of 0.003, and *p* = 0.001; Supplementary Figure S4E).

*6. Explantation of Perceval Bioprosthesis*

Only two studies reported the explantation rate of the Perceval bioprosthesis 5 to 10 years post-SUAVR, showing an explantation rate due to endocarditis of 1.12% (15 of 1331 cases with an estimated rate of 1.0% and 95% CI: 0.4% to 1.7%, SE of 0.003, and *p* = 0.002; Supplementary Figure S4F).

*7. Comparison of Mid-term and Long-term Outcomes*

**Figure 7:**
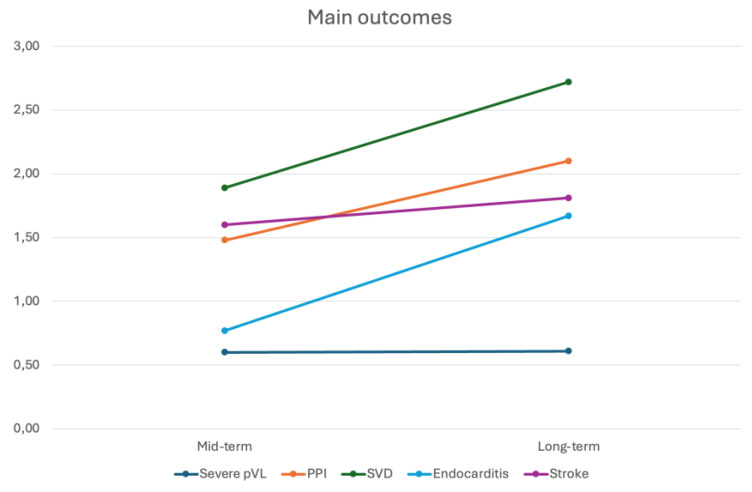
The occurrence of serious complications between the mid-term and long-term follow-up periods after SUAVR.

The occurrence of serious complications such as severe PVL, PPI, SVD, endocarditis, and stroke did not differ significantly between the mid-term and long-term follow-up periods after SUAVR (Figure 7). Overall, the incidence of these serious complications following SUAVR can be considered as low.

*Mid-Term and Long-Term Echocardiographic Findings After SUAVR*

**Figure 8:**
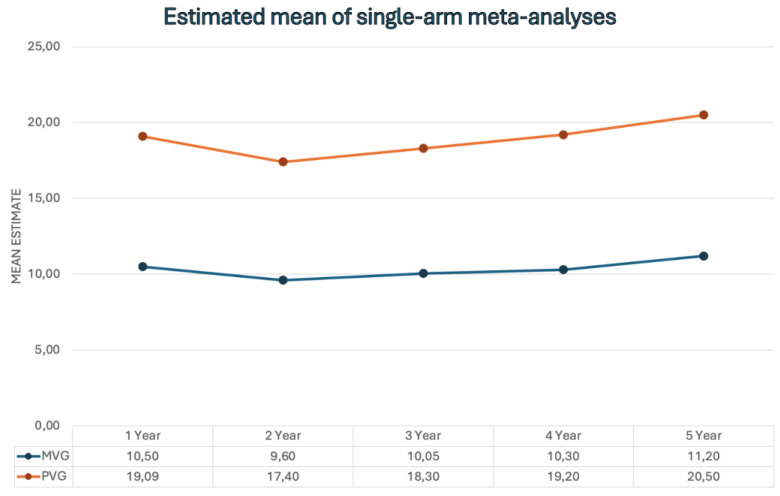
Mid-term outcomes following SUAVR.

According to the single-arm meta-analyses, the estimated mean values and 95% confidence intervals for MVG and at 1 to 5 years following SUAVR are provided in Supplementary Table S11. However, the report notes that the data on echocardiographic findings during the long-term follow-up period were not sufficiently reported. Therefore, an analysis of the long-term echocardiographic outcomes was not possible (Figure 8).

*Life Expectancy After SUAVR*

**Figure 9:**
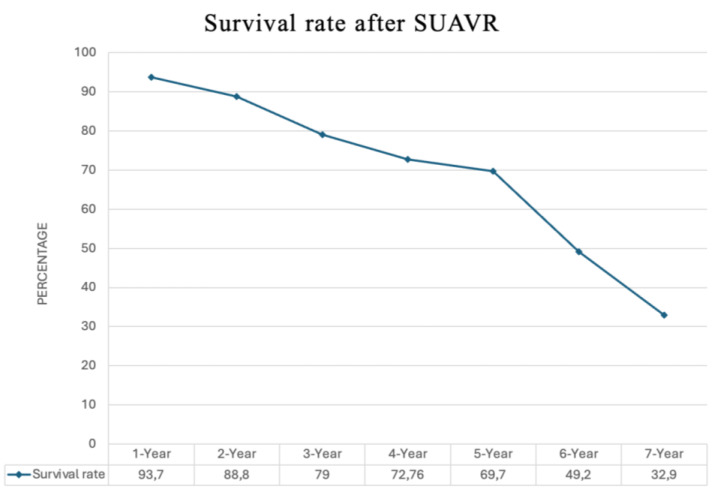
Life expectancy after SUAVR.

The reported overall survival rates following SUAVR showed a gradual decline over time. The first year’s survival rate was 93.7% (4572 out of 4876 cases), which decreased to 88.8% (3353 out of 3774 cases) in the second year, 79% (2357 out of 2995 cases) in the third year, 72.7% (2397 out of 3294 cases) in the fourth year, and 69.7% (3135 out of 4493 cases) in the fifth year. The survival rate further dropped to 49.2% (1038 out of 2106 cases) in the sixth year and 32.9% (547 out of 1660 cases) in the seventh year (Figure 9). However, the report notes that the data on cases that survived beyond the eighth year were not sufficiently reported. Therefore, an analysis of survival rates for years more than the seventh year was not possible based on the available information. The single-arm meta-analyses on survival rates are detailed in Supplementary Figure S5A–G.

## 4. Discussion

Our meta-analysis provided additional insights into the comparison between SUAVR and TAVI, demonstrating that patients treated with SUAVR experience significantly lower rates of mild and moderate to severe PVL compared to TAVI. While SUAVR was associated with a higher mean and MVG, the subgroup analysis of minimally invasive SUAVR (MI-SUAVR) showed no significant difference in MVG compared to TAVI. No statistically significant differences were observed in PPI rates between the two groups. SUAVR showed a lower incidence of NOMI but was associated with higher rates of NOAF and major bleeding. Conversely, TAVI had higher rates of LBBB and major vascular complications. Although pooled results did not reveal statistically significant differences in 30-day mortality, we observed a trend towards better mid-term survival in the SUAVR group. 

This meta-analysis represents the first comprehensive comparison of SUAVR versus TAVI without including rapid deployment aortic valve replacement (RDAVR), aiming to provide more specific insights into the outcomes of these two procedures. A previous meta-analysis compared TAVI with SUAVR and RDAVR, demonstrating a lower risk of PVL with SU/RDAVR and a higher mortality with TAVI [38]. In comparison between SUAVR and RDAVR, SUAVR tended to have shorter ACC and CPB times, while RDAVR showed advantages in terms of PVG and MVG [39,40]. SU/RDAVR exhibited similar clinical and hemodynamic behaviors, demonstrating comparable outcomes. The duration of ACC has been identified as a critical and independent risk predictor of severe cardiovascular morbidities [41]. Furthermore, prolonged ACC time significantly increases the rates of postoperative morbidity, including AKI and multiorgan failure, in patients undergoing AVR [42]. Therefore, the Perceval valve may offer substantial benefits for “gray zone” patients, who are at high risk for conventional SAVR but are operable by reduced ACC and CPB times.

Additionally, minimally invasive and endoscopic approaches through mini-right thoracotomy have been becoming a feasible option. The Perceval bioprosthesis allows the valve to collapse on its holder and maximizes visualization of the aortic annulus during positioning and deployment, making it a better option in such scenarios [40]. Given this context, our study focuses exclusively on comparing SUAVR and TAVI, excluding RDAVR, to provide a clearer comparison. 

Our meta-analysis demonstrated a significantly lower rate of PVL associated with SUAVR compared to TAVI (OR: 0.05; 95% CI: 0.03 to 0.08; *p* < 0.00001), for both mild and moderate-to-severe cases (OR: 0.14; 95% CI: 0.07 to 0.28; *p* < 0.00001). This is a crucial observation, as even mild PVL has been associated with increased mortality [43,44]. The lower rates of PVL observed with SUAVR can be attributed to several factors. Firstly, the thorough removal of calcified valve leaflets during SUAVR, even when using sutureless techniques, likely contributes to the better fitting of the valve [45]. This meticulous preparation of the annulus reduces the risk of residual calcification interfering with valve seating. Additionally, placing the valve with direct visual or endoscopic guidance in SUAVR allows for accurate deployment, ensuring optimal seating and reducing gaps that could lead to PVL [46]. In contrast, TAVI, which is deployed through a catheter, may be prone to placement variability and challenges in fully apposing the valve to the native annulus, contributing to higher PVL rates. Given the critical impact of PVL on long-term outcomes, the superior results of SUAVR highlight a significant advantage in terms of patient prognosis. The better trend of mid-term survival in the SUAVR group (85.2% vs. 78.9%, OR: 1.89; 95% CI: 0.98 to 3.65; *p* = 0.06) could be explained by the higher incidence of any level of PVL in TAVI group. 

In our meta-analysis, SUAVR was associated with a higher early MVG than TAVI, with an MD of 2.27 mmHg (95% CI: 1.0 to 3.54; *p* = 0.0004). This aligns with prior studies comparing SU/RDAVR and TAVI, which reported an MD of 1.59 mmHg, suggesting minimal clinical impact given the lack of significant AVA differences [38]. The higher gradients in SUAVR can be influenced by procedural factors like postoperative anemia, hemodilution, oversized valves, and inflammation [15,47]. However, our subgroup analysis of MI-SUAVR versus TAVI found no significant difference in early MVG (MD: 0.37, 95% CI: −1.05 to 1.80, *p* = 0.61). 

MI-SUAVR has shown benefits over full-median sternotomy SUAVR, including shorter ventilation times, reduced hospital stays, and lower NOAF rates [48]. Therefore, MI-SUAVR might be particularly advantageous for intermediate to high-risk patients who are unsuitable for TAVI, maximizing valve performance with reduced procedural invasiveness and thus optimizing outcomes. It was found that the incidence of NOAF was significantly higher in patients undergoing SUAVR than TAVI. However, NOAF can be effectively treated with appropriate therapies in the perioperative period. Kondo et al. reported a technique for simultaneous resection of the left atrial appendage performed during minimally invasive aortic valve replacement via the right anterolateral thoracotomy through the transverse sinus [49]. By elevating the collapsed ascending aorta, the left atrial appendage could be safely exposed and resected with a surgical stapler. This simultaneous approach may be a viable option, particularly in elderly patients for whom a percutaneous procedure is unsuitable, and could help prevent thromboembolic and hemorrhagic complications associated with the left atrial appendage.

Our study showed a PPI rate of 7.27% in the SUAVR group, compared to 10.3% in the TAVI group. The pooled analysis indicated no statistical difference in the PPI rate between the two groups but showed a higher trend of PPI rate in the TAVI group (OR 0.76; 95% CI: 0.48−1.19; *p* = 0.23). Notably, TAVI exhibited a higher risk of LBBB with an incidence of 27.4% compared to 18.4% in the SUAVR group (OR: 0.60; 95% CI: 0.38−0.92; *p* = 0.02), suggesting greater stress on the conduction pathways. The implantation technique of the Perceval valve initially recommended placing the guide sutures 2–3 mm beneath the leaflet insertion line. According to Yanagawa et al., the PPI rate was reduced from 28% with the initial recommended technique to 0% by modifying the suture placement to the nadir of the aortic annulus [50]. The transcatheter valve is deployed under rapid or controlled pacing, making fine adjustments in depth and angle challenging due to calcified leaflets and blood flow effects.

In contrast, SUAVR is performed under cardioplegic arrest, allowing for precise placement with guide sutures, which theoretically limits deeper implantation. The initial learning curve for the proper implantation technique might be needed for SUAVR, and this method can offer consistent and reproducible placement. These differences in implantation techniques may contribute to the slight variance in PPI rates observed between SUAVR and TAVI. In TAVI, factors such as valve oversizing, post-dilation, and deep implantation have been reported as risks for PPI [51,52]. Similarly, efforts should be made to avoid these risk factors in SUAVR to minimize the risk for PPI.

As TAVI expands its indications to younger and lower-risk patients, it is crucial to evaluate long-term durability and reoperation strategies for “gray zone” patients undergoing AVR. Our study estimated the mid-term SVD rate for SUAVR to be 1.4% and the long-term rate of 2.7%, indicating sustained durability. SUAVR also showed low explantation rates (1.64% within 1 to 5 years). No significant differences in complications such as severe PVL, PPI, SVD, and stroke were observed between mid-term and long-term follow-ups. Fukuhara et al. highlighted that 71% of TAVI failures required explantation due to anatomical challenges, with a high 30-day mortality rate of 15%, underscoring the risks of transcatheter valve explantation [53]. The Perceval valve’s design, its radio-opaque frame, and struts that keep coronary ostia and sinuses away from Perceval valve leaflets might simplify future valve-in-valve TAVI procedures, providing a strategic advantage for “gray zone” patients [46]. Therefore, SUAVR can offer a robust, durable solution, balancing immediate procedural benefits with favorable long-term outcomes and reduced reoperation risks, making it a suitable choice for those expected to have prolonged survival and a higher likelihood of requiring future interventions. Several limitations must be considered when interpreting the results of this meta-analysis. First, the analysis included studies with propensity score matching, none of which were randomized controlled trials. Second, considerable heterogeneity was observed in some analyses, and detailed descriptions of implantation techniques, which could impact results, were not well-described. Third, while the SUAVR group consisted solely of patients with the Perceval valve, the TAVI group included a mix of balloon-expandable and self-expandable valves, each with different implantation techniques, hemodynamic characteristics, and complication rates. The granularity of information on these differences was insufficient. We recommend that future studies provide more specific definitions and subtypes of outcomes, as well as more detailed reporting of demographic differences, to allow better evaluation of outcomes.

## 5. Conclusions

Our meta-analysis shows that SUAVR has a lower incidence of PVL and a favorable mid-term overall survival compared to TAVI. Despite the need for further studies to comprehensively compare the long-term clinical outcomes of SUAVR and TAVI, these findings highlight SUAVR as a valuable, promising option for “gray zone” patients. Deciding the optimal surgical approach should involve careful consideration of each patient’s comorbidities, anatomical suitability, and the potential risks of reoperation.

## Figures and Tables

**Table 1:** Characteristics of included articles for the first part of the study.

First Author	Year	Study Period	Country	Surgical Center	Study Type	Median Follow-Up (Months)	Propensity-Machted
Vilalta et al. [12]	2021	2011–2020	Canada/Spain	Multicenter	Prospective non-randomized	24 (12–36)	Yes
Chung et al. [13]	2021	2011–2019	Republic of Korea	Single-Center	Non-prospective non-randomized	12.9 (4.1–26.5)	Yes
Gerfer et al. [14]	2021	2012–2017	Germany	Single-Center	Non-prospective non-randomized	ND	Yes
Dónofrio et al. [15]	2016	2007–2012	Italy	Multicenter	Non-prospective non-randomized	At least 1 Year	Yes
Miceli et al. [16]	2016	2008–2013	Italy	Single-Center	Non-prospective non-randomized	13 (7–25)	Yes
Muneretto et al. [17]	2020	2008–2015	Italy	Multicenter	Non-prospective non-randomized	60 months	Yes
Biancari et al. [23]	2016	2007–2014	Itay/Germany/Sweden/Belgium	Multicenter	Non-prospective non-randomized	Until Discharge	Yes
Zubarevich et al. [19]	2022	2018–2021	Germany	Single-Center	Non-prospective non-randomized	18.1 ± 12.3	Yes
Santarpino et al. [20]	2022	2010–2018	Italy	Multicenter	Non-prospective non-randomized	42.87 ± 21.69	Yes
Santarpino et al. [21]	2015	2010–2015	Germany	Single-Center	Randomized non-prospective	24.5 ± 13.8	Yes
Indelen et al. [22]	2023	2015–2020	Turkey	Single-Center	Non-prospective non-randomized	ND	Yes
Muneretto et al. [18]	2023	2013–2020	Italy/Germany/France	Multicenter	Non-prospective non-randomized	51.6 (13.2–88.8)	Yes

**Table 2:** Characteristics of included articles for the second part of the study.

First Name	Year	County	Center	Study Period	Type of Study	Sample Size	Median-Follow-UP (Year)
Aldea et al. [24]	2023	USA	Multicenter	2010–2015	Retrospective observational study	970	4 years
Concistre et al. [25]	2023	Italy	Single-Center	2011–2021	Prospective cohort study	1652	1 year (up to 8 years)
Dokollari et al. [26]	2023	The Netherlands	Single-Center	2013–2020	Retrospective observational study	101	7 years
Ferreira et al. [27]	2022	Portugal	Single-Center	2015–2020	Retrospective observational study	196	Up to 5 years
Fischlein et al. [28]	2021	Germany	Multicenter	2010–2013	Prospective cohort study	658	3.8 years
Glauber et al. [29]	2020	Italy	Multicenter	2011–2018	Prospective cohort study	480	2.4 years
Hong et al. [30]	2024	Korea	Single-Center	2015–2020	Retrospective observational study	113	51.19 ± 20.6
Kapadia et al. [31]	2024	UK	Single-Center	2014–2020	Retrospective observational study	102	ND
Lamberigts et al. [32]	2022	Belgium	Single-Center	2007–2019	Retrospective observational study	784	7.03 years
Meuris et al. [33]	2015	Belgium	Multicenter	2007–2008	Prospective cohort study	30	4.2 years
Muneretto et al. [17]	2022	Italy	Multicenter	2008–2015	Retrospective observational study	481	5 years
Pollari et al. [34]	2023	Germany	Single-Center	2010–2020	Retrospective observational study	547	4.53 years
Santarpino et al. [20]	2022	Italy	Multicenter	2010–2018	Retrospective observational study	172	6.1 years
Schizas et al. [35]	2024	Greece	Single-Center	2013–2020	Retrospective observational study	205	6.27 ± 2.03
Szecel et al. [36]	2021	Belgium	Single-Center	2007–2017	Retrospective observational study	468	3.1 ± 2 up to 11.2 year
White et al. [37]	2022	Canada	Single-Center	2013–2019	Retrospective observational study	295	2.4 years

## Data Availability

The data are available from the corresponding author upon reasonable request.

## Supplementary Materials

The following supporting information can be downloaded at: https://www.mdpi.com/article/10.3390/jcm13164887/s1, Table S1. Information about SUAVR for the first part of the study, Table S2. Information about TAVI for the first part of the study, Table S3. Demographic Data of included articles for the first part of the study, Table S4. Echocardiographic Data of included articles for the first part of the study, Table S5. Reported clinical outcomes and complications after SUAVR or TAVI of included articles for the first part of the study, Table S6. Reported clinical outcomes and complications after SUAVR or TAVI of included articles for the first part of the study (Continue 1), Table S7. Reported clinical outcomes and complications after SUAVR or TAVI of included articles for the first part of the study (Continue 2), Table S8. Demographic Data of included articles for the second part of the study, Table S9. Information about surgical related demographics for the second part of the study, Table S10. Information about SUAVR for the second part of the study, Table S11. Echocardiographic Data of included articles for the second part of the study, Figure S1. Subgroup analysis based on minimally invasive SUAVR, Figure S2. Comparison of mid-term outcomes between SUAVR and TAVI, Figure S3. Explantation of Perceval Bioprosthesis at mid-term follow-up, Figure S4. Long-term Outcomes Following SUAVR, Figure S5. Life expectancy after SUAVR.
