# Supplementary material for: Sutureless Aortic Valve Replacement with Perceval Bioprosthesis Superior to Transcatheter Aortic Valve Implantation: A Promising Option for the Gray-Zone of Aortic Valve Replacement Procedures—A State-of-the-Art Systematic Review, Meta-Analysis, and Future Directions
Source: J Clin Med. 2024 Aug 19;13(16):4887. doi: 10.3390/jcm13164887 (PMC11355092; doi:10.3390/jcm13164887)

Supplementary Materials. Tables

Table S1. Information about SUAVR for the first part of the study.

First Author	Year	Full-Sternotomy	MICS	MS	MT	Valve Size mean/SD	Size: S	Size: M	Size: L	Size: XL	CPB-Time min	ACC Time min
Vilalta et al.	2021	96 (56.1%)	75 (43.9%)	ND	ND	23 ± 1.7	ND	ND	ND	ND	ND	ND
Chung et al.	2021	ND	ND	ND	ND	ND	ND	ND	ND	ND	72.6 ± 24.8	39.9 ± 14.2
Gerfer et al.	2021	29 (49%)	30 (51%)	51 (86%)	8 (14%)	24 ± 2	4 (7%)	27 (46%)	17 (29%)	11 (19%)	83 ± 32	49 ± 22
Dónofrio et al.	2016	ND	ND	ND	ND	ND	ND	ND	ND	ND	ND	ND
Miceli et al.	2016	0 (0%)	37 (100%)	0 (0%)	37 (100%)	ND	ND	ND	ND	ND	ND	ND
Muneretto et al.	2020	ND	ND	ND	ND	23 ± 1	ND	ND	ND	ND	54 ± 25	32 ± 14
Biancari et al.	2016	174 (45.9%) of all patients before Matching	205 (54.1%) of all patients before Matching	ND	ND	ND	ND	ND	ND	ND	ND	ND
Zubarevich et al.	2022	ND	ND	ND	ND	ND	8 (14.3%)	11 (19.6%)	16 (28.6%)	22 (39.2%)	49.4 ± 17.4	ND
Santarpino et al.	2022	40 (23.3%)	132 (76.7%)	ND	ND	22.91 ± 2.73	ND	ND	ND	ND	80.63 ± 45.5	59.56 ± 29.2
	2015	36%	64.4%	61%	3.4%	ND	ND	ND	ND	ND	ND	ND

Santarpino et al.												
Indelen et al.	2023	ND	ND	ND	ND	ND	ND	ND	ND	ND	117.56	76.93
Muneretto et al.	2023	31.7%	68.3%	53.6%	14.7%	23.5 ± 0.57	ND	ND	ND	ND	ND	ND

Table S2. Information about TAVI for the first part of the study.

First Author	Year	TAVI-Access	BEV	SEV	MEV	Valve Size mean/SD	Size: S	Size: M	Size: L	Size: XL
Vilalta et al.	2021	TF: 77.2%, TC: 18.1%, TAX: 3.5%, TAO: 1.2%	99 (57.9%)	67 (39.2%)	5 (2.9%)	26 ± 3	ND	ND	ND	ND
Chung et al.	2021	TF: 98%, Transsubclavian: 1.2%, TAO: 0.8%	15 (24.2%)	44 (70.9%)	3 (4.8%)	23.8 ± 2.2	ND	ND	ND	ND
Gerfer et al.	2021	TF: 100%	0 (0%)	59 (100%)	0 (0%)	25 ± 2	21 (36%)	23 (39%)	15 (25%)	ND
Dónofrio et al.	2016	TF: 66.2%, TA:33.8%	214 (100%)	0 (0%)	0 (0%)	ND	ND	ND	ND	ND
Miceli et al.	2016	TF: 51.6%, TA: 48.4%	37 (100%)	0 (0%)	0 (0%)	ND	ND	ND	ND	ND
Muneretto et al.	2020	TF: 71.1%, Non-TF: 28.8%	101 (34.7%)	190 (65.2%)	0 (0%)	25.5 ± 1.11	ND	ND	ND	ND
Biancari et al.	2016	TF: 97.7%, Subclaviean: 0.8%, Tansapical: 1% and transaortic: 0.5%	ND	ND	ND	ND	ND	ND	ND	ND
Zubarevich et al.	2022	TA: 100%	56 (100%)	ND	ND	ND	18 (32.3%)	28 (50%)	9 (16%)	0 (0%)
Santarpino et al.	2022	TF: 68%, Other Access:32%	0 (0%)	172 (100%)	0 (0%)	ND	ND	ND	ND	ND
Santarpino et al.	2015	TF: 56%, Transapical: 43%, Transaortic: 0.2%	ND	ND	ND	ND	ND	ND	ND	ND
Indelen et al.	2023	TF: 100%	ND	ND	ND	ND	ND	ND	ND	ND
Muneretto et al.	2023	TF: 79.4%, Transapical: 15.2%, Other Access:5.3%	18.3%	81.7%	ND	26 ± 1.7	ND	ND	ND	ND

Table S3. Demographic Data of included articles for the first part of the study.

First Author	Year	Intervention Groups	Sample Size	Gender (N)	Mean Age	Euroscore	DM	HTN	CKD	COPD	Prior MI	PAD	Redo	NYHA I	NYHA II	NYHA III		NYHA IV	AS	AR
Vilalta et al.	2021	SuAVR Perceval	171	64 (37.4%)	78 ± 5.7	1.9 ± 0.4	52 (30.4%)	144 (84.2%)	ND	28 (16.4%)	13 (7.6%)	14 (8.2%)	7 (4.1%)	ND	ND	80 (46.8%)		100%	0%	Low
		TAVI	171	62 (36.3%)	77.4 ±8.4	1.9 ± 0.4	52 (34.5%)	144 (84.2%)	ND	22 (12.9%)	14 (8.2%)	12 (7%)	10 (5.9%)	ND	ND	84 (49.1%)		100%	0%	
Chung et al.	2021	SuAVR Perceval	62	24 (38.7%)	75.5 ± 5.3	More than 4%: 8 (12.9%)	24 (38./%)	52 (83.9%)	25 (40.3%)	8 (12.9%)	ND	4 (6.5%)	5 (8.1%)	ND	ND	39 (62.9%)		100%	0%	High
		TAVI	62	20 (32.3%)	76.8 ± 6	More than 4%: 9 (14.5%)	23 (37.1%)	54 (87.1%)	26 (41.9%)	8 (12.9%)	ND	7 (11.3%)	4 (6.5%)	ND	ND	37 (59.7%)		100%	0%	
Gerfer et al.	2021	SuAVR Perceval	59	21 (36%)	77 ± 8	2.5 ± 1.2	18 (31%)	55 (93%)	20 (34%)	4 (7%)	11 (19%)	10 (17%)	2 (3%)	ND	ND	ND	ND	100%	0%	moderate
		TAVI	59	21 (36%)	79 ± 5	2.5 ± 1.2	16 (27%)	51 (86%)	22 (37%)	13 (22%)	6 (10%)	11 (19%)	3 (5%)	ND	ND	ND	ND	100%	0%	
Dónofrio et al.	2016	SuAVR Perceval	214	76 (35.5%)	77.4 ± 5.4	10.5± 6.2	59 (27.6%)	190 (88.8%)	168 (78.5%)	39 (18.2%)	12 (5.6%)	46 (21.5%)	22 (10.8%)	5 (2.3%)	60 (28%)	134 (62.6%)	15 (7%)	100%	0%	High
		TAVI	214	75 (35%)	77.7 ± 7.9	12.4 ± 9.1	58 (27.1%)	160 (74.8%)	172 (80%)	36 (16.8%)	11 (5.1%)	48 (22.4%)	21 (9.8%)	5 (2.3%)	52 (24.3%)	135 (63.1%)	22 (10.3%)	100%	0%	
Miceli et al.	2016	SuAVR Perceval	37	13 (30%)	79 ± 4.5	16.1 ± 11	10 (27%)	32 (86.5%)	ND	8 (21.6%)	ND	11 (29.7%)	3 (8.1%)	ND	ND	25 (67.6%)		100%	0%	High
		TAVI	37	15 (40.5%)	78.8 ± 7.4	15.7 ± 8.5	7 (18.9%)	31 (83.8%)	ND	11 (29.7%)	ND	9 (24.3%)	3 (8.1%)	ND	ND	22 (59.5%)		100%	0%	
Muneretto et al.	2020	SuAVR Perceval	291	121 (41.5%)	80 ± 5	14.15 ± 1.75	61 (20.9%)	224 (77%)	52 (17.9%)	63 (21.6%)	20 (6.9%)	53 (18.2%)	13 (4.5%)	ND	ND	206 (70.8%)		100%	0%	Moderate
		TAVI	291	121 (41.5%)	81 ± 6	13.9 ± 1.6	60 (20.6%)	227 (78%)	67 (23%)	69 (23.7%)	16 (5.5%)	51 (17.5%)	12 (4.1%)	ND	ND	209 (71.8%)		100%	0%	
Biancari et al.	2016	SuAVR Perceval	144	56 (38.8%)	79.4 ± 5.4	4.1 ± 3.2	ND	ND	113 (78.4%)	38 (26.4%)	5 (3.5%)	12 (8.3%)	12 (8.3%)	ND	ND	108 (75%)		ND	ND	Moderate-High (more high Risk in TAVI Group)
		TAVI	144	54 (37.5%)	79 ± 6	3.6 ± 2.6	ND	ND	121 (84%)	35 (24.3%)	3 (2.1%)	13 (9%)	15 (10.4%)	ND	ND	105 (72.9%)		ND	ND	

Zubarevich et al.	2022	SuAVR Perceval	56	43 (76.7%)	69.05 ± 7.9	3.07 ± 1	19 (33.9%)	54 (96.4%)	13 (23.3%)	11 (19.6%)	8 (14.3%)	9 (16.1%)	5 (8.9%)	4 (7.1%)	33 (58.9%)	20 (35.7%)	ND	52 (92.9%)	10 (17.9%)	Moderate
		TAVI	56	42 (75%)	74.3 ± 7.8	3.5 ± 0.9	20 (35.7%)	52 (92.9%)	17 (30.4%)	15 (26.8%)	15 (26.8%)	29 (51.8%)	1 (1.8%)	14 (25%)	21 (37.5%)	21 (37.5%)	ND	56 (100%)	0%	
Santarpino et al.	2022	SuAVR Perceval	172	67 (38.9%)	80.9 ± 5.1	5.6 ± 2.9	24 (13.9%)	99 (57.5%)	62 (36%)	75 (43.6%)	25 (14.5%)	22 (12.7%)	15 (8.7%)	ND	ND	ND	ND	100%	0%	High (more high Risk in TAVI Group)
		TAVI	172	74 (43%)	79.1 ± 7.4	6.1 ± 1.5	32 (18.6%)	114 (66.2%)	69 (40.1%)	69 (40.1%)	42 (24.4%)	31 (18%)	26 (15.1%)	ND	ND	ND	ND	100%	0%	
Santarpino et al.	2015	SuAVR Perceval	102	42 (41%)	80 ± 4	17 ± 14	40 (39%)	88 (86%)	34 (33%)	22 (22%)	1 (1%)	27 (26%)	13 (13%)	ND	ND	ND	ND	100%	0%	High
		TAVI	102	44 (43%)	79 ± 7	18 ± 11	37 (36%)	94 (92%)	33 (32%)	24 (23%)	1 (1%)	16 (16%)	14 (14%)	ND	ND	ND	ND	100%	0%	
Indelen et al.	2023	SuAVR Perceval	61	31 (50.8%)	72.33	2.72	18 (29.6%)	27 (44.3%)	5 (8.2%)	5 (8.2%)	9 (14.8%)	9 (14.8%)	7 (11.5%)	34 (55.7%)	19 (31.1%)	6 (9.8%)	2 (3.3%)	100%	0%	moderate
		TAVI	53	24 (45.3%)	78	3.34	9 (16.9%)	20 (37.7%)	5 (9.4%)	5 (9.4%)	4 (7.5%)	6 (11.3%)	10 (18.9%)	26 (49.1%)	21 (39.6%)	5 (9.4%)	1 (1.9%)	100%	0%	
Muneretto et al.	2023	SuAVR Perceval	517	194 (37.5%)	81 ± 1.7	5.6 ± 0.7	160 (30.9%)	424 (82%)	22 (4.3%)	114 (22%)	74 (14.3%)	125 (24.2%)	43 (8.3)	ND	ND	347 (67.1%)		100%	0%	Moderate
		TAVI	517	198 (38.2%)	81.6 ± 2.1	5.7 ± 0.9	165 (31.9%)	432 (83.6%)	22 (4.3%)	99 (19.2%)	68 (13.1%)	129 (24.9%)	45 (8.7%)	ND	ND	343 (66.3%)		100%	0%	

Table S4. Echocardiographic Data of included articles for the first part of the study.

First Author	Year		Sample Size	Procedure Time (min)		MVG (mmHg)		PVG (mmHg)	
				Mean	SD	Mean	SD	Mean	SD
Vilalta et al.	2021	SuAVR Perceval	171	ND	ND	16.8	6.9	ND	ND
		TAVI	171	ND	ND	11.4	5.7	ND	ND
Chung et al.	2021	SuAVR Perceval	62	ND	ND	14.7	3.8	27.5	5
		TAVI	62	ND	ND	11.2	5.7	21.4	10.5
Gerfer et al.	2021	SuAVR Perceval	59	ND	ND	12	4	ND	ND
		TAVI	59	ND	ND	9	4	ND	ND
Dónofrio et al.	2016	SuAVR Perceval	214	ND	ND	13.7	6.6	26.7	12.1
		TAVI	214	ND	ND	11	4.6	20.3	8.1
Miceli et al.	2016	SuAVR Perceval	37	ND	ND	11.4	3.7	19.2	6.9
		TAVI	37	ND	ND	10.1	3.4	19.7	5.4
Muneretto et al.	2020	SuAVR Perceval	291	ND	ND	10.6	6.2	20.9	9.5
		TAVI	291	ND	ND	12.8	6.5	22.9	10
Biancari et al.	2016	SuAVR Perceval	144	ND	ND	ND	ND	ND	ND
		TAVI	144	ND	ND	ND	ND	ND	ND
Zubarevich et al.	2022	SuAVR Perceval	56	149.05	48.3	5.8	0.8	ND	ND
		TAVI	56	67.3	34.7	5	0.6	ND	ND
Santarpino et al.	2022	SuAVR Perceval	172	ND	ND	ND	ND	ND	ND
		TAVI	172	ND	ND	ND	ND	ND	ND
Santarpino et al.	2015	SuAVR Perceval	102	ND	ND	ND	ND	ND	ND
		TAVI	102	ND	ND	ND	ND	ND	ND
Indelen et al.	2023	SuAVR Perceval	61	ND	ND	ND	ND	ND	ND
		TAVI	53	ND	ND	ND	ND	ND	ND
Muneretto et al.	2023	SuAVR Perceval	517	ND	ND	10.6	6.2	21.8	6.5
		TAVI	517	ND	ND	10.8	6.5	22.4	6.4

Table S5. Reported clinical outcomes and complications after SUAVR or TAVI of included articles for the first part of the study.

First Author	Year	Intervention Groups	Sample Size	PVL any	PVL Mild	PVL Mod-Severe	PPI	PPM	Atrial Fibrilation	Coronary Obstruction	LBBB	Stroke	AKI	Dialysis	MI	Major-Bleeding
Vilalta et al.	2021	SuAVR Perceval	171	ND	ND	1 of 159 (0.6%)	15 (8.8%)	12 of 26 (46.2%)	56 (32.8%)	ND	28 (16.4%)	4 (2.3%)	ND	ND	0 (0%)	98 (57.3%)
		TAVI	171	ND	ND	1 of 94 (1.1%)	31 (18.1%)	49 of 159 (30.8%)	7 (4.1%)	ND	43 (25.2%)	5 (2.9%)	ND	ND	2 (1.2%)	7 (4.1%)
Chung et al.	2021	SuAVR Perceval	62	5 (8.1%)	5 (8.1%)	0 (0%)	4 (6.5%)	8 (12.9%)	20 (32.3%)	0 (0%)	15 (24.2%)	3 (4.8%)	13 (21%)	1 (1.6%)	0 (0%)	14 (22.6%)
		TAVI	62	37 (59.6%)	36 (58.1%)	1 (1.6%)	9 (14.5%)	6 (9.7%)	3 (4.8)	1 (0.4%)	21 (33.9%)	2 (3.2%)	5 (8.1%)	0 (0%)	0 (0%)	6 (9.7%)
Gerfer et al.	2021	SuAVR Perceval	59	0 (0%)	0 (0%)	0 (0%)	6 (10.2%)	ND	ND	ND	ND	3 (5.1%)	ND	ND	0 (0%)	1 (1.7%)
		TAVI	59	37 (62.7%)	33 (55.9%)	4 (6.8%)	5 (8.5%)	ND	ND	ND	ND	1 (1.7%)	ND	ND	1 (1.7%)	0 (0%)
Dónofrio et al.	2016	SuAVR Perceval	214	6 (2.8%)	5 (2.3%)	1 (0.5%)	20 (9.4%)	ND	ND	ND	ND	4 (1.9%)	ND	ND	1 (0.5%)	40 (18.7%)
		TAVI	214	73 (34.1%)	62 (30%)	11 (5.3%)	6 (2.8%)	ND	ND	ND	ND	4 (1.9%)	ND	ND	2 (0.9%)	34 (16.1%)
Miceli et al.	2016	SuAVR Perceval	37	2 (5.4%)	2 (5.4%)	0 (0%)	2 (5.4%)	ND	ND	ND	ND	0 (0%)	11 (6.2%)	ND	ND	1 (2.7%)
		TAVI	37	30 (81.1%)	20 (54%)	10 (27%)	0 (0%)	ND	ND	ND	ND	3 (8.1%)	4 (4.4%)	ND	ND	1 (2.7%)
Muneretto et al.	2020	SuAVR Perceval	291	ND	ND	3 (1%)	16 (5.5%)	0 (0%)	ND	0 (0%)	ND	6 (2.1%)	9 (3.1%)	3 (1%)	1 (0.3%)	14 (4.8%)
		TAVI	291	ND	ND	17 (5.8%)	31 (10.7%)	0 (0%)	ND	1 (0.3%)	ND	10 (3.4%)	19 (6.5%)	8 (2.7%)	5 (1.7%)	2 (0.1%)
Biancari et al.	2016	SuAVR Perceval	144	4 (2.8%)	3 (2.1%)	1 (0.7%)	16 (11.2%)	ND	ND	ND	ND	0 (0%)	ND	3 (2.1%)	ND	6 (4.2%)
		TAVI	144	76 (53.5%)	55 (38.7%)	21 (14.8%)	22 (15.4%)	ND	ND	ND	ND	3 (2.1%)	ND	0 (0%)	ND	0 (0%)
Zubarevich et al.	2022	SuAVR Perceval	56	0 (0%)	0 (0%)	0 (0%)	0 (0%)	ND	ND	ND	ND	0 (0%)	ND	5 (8.9%)	ND	31 (55.4%)
		TAVI	56	0 (0%)	0 (0%)	0 (0%)	1 (1.8%)	ND	ND	ND	ND	1 (1.8%)	ND	3 (5.4%)	ND	6 (10.7%)

Santarpino et al.	2022	SuAVR Perceval	172	ND	ND	ND	10 (5.8%)	ND	53 (30.8%)	ND	ND	ND	ND	ND	ND	59 (34.3%)
		TAVI	172	ND	ND	ND	20 (11.6%)	ND	9 (5.2%)	ND	ND	ND	ND	ND	ND	16 (9.3%)
Santarpino et al.	2015	SuAVR Perceval	102	7 (7%)	ND	ND	10 (10%)	ND	ND	ND	ND	3 (2.9%)	5 (4.9%)	ND	ND	ND
		TAVI	102	35 (34%)	ND	ND	9 (9.4%)	ND	ND	ND	ND	5 (4.9%)	1 (1%)	ND	ND	ND
Indelen et al.	2023	SuAVR Perceval	61	0 (0%)	0 (0%)	0 (0%)	0 (0%)	ND	ND	ND	ND	3 (4.9%)	4 (6.5%)	ND	ND	ND
		TAVI	53	0 (0%)	0 (0%)	0 (0%)	0 (0%)	ND	ND	ND	ND	2 (3.7%)	5 (9.4%)	ND	ND	ND
Muneretto et al.	2023	SuAVR Perceval	517	ND	ND	5 (0.97%)	33 (6.4%)	31 (5.9%)	ND	ND	ND	8 (1.5%)	11 (2.1%)	5 (1)	11 (2.1%)	21 (4.1%)
		TAVI	517	ND	ND	25 (4.8%)	61 (11.8%)	43 (8.3%)	ND	ND	ND	14 (2.7%)	23 (4.4%)	12 (2.3%)	23 (4.4)	10 (1.9%)

Table S6. Reported clinical outcomes and complications after SUAVR or TAVI of included articles for the first part of the study (Continue 1).

First Author	Year	Intervention Groups	Sample Size	Conversion	Annulus Rupture	Vascular Complications	ICU-Stay		Hospital-Stay (h)		Postoperative Aortic-Valve-Area cm2		Device Sucess	In-hospital-Mortality	30-day-Mortality
							Mean	SD	Mean	SD	Mean	SD			
Vilalta et al.	2021	SuAVR Perceval	171	ND	0 (0%)		ND	ND	ND	ND	1.5	0.23	ND	7 (4.1%)	ND
		TAVI	171	ND	0 (0%)		ND	ND	ND	ND	1.8	0.21	ND	3 (1.8%)	ND
Chung et al.	2021	SuAVR Perceval	62	ND	ND	0 (0%)	5.9	9.2	13.1	8.8	1.6	0.4	49 (79%)	ND	0 (0%)
		TAVI	62	ND	ND	2 (3.2%)	1.9	1.6	7.1	7.9	1.8	0.4	49 (79%)	ND	3 (4.8%)
Gerfer et al.	2021	SuAVR Perceval	59	ND	ND	ND	4	5	12	5	ND	ND	ND	ND	3 (5.1%)
		TAVI	59	ND	ND	ND	3	3	9	5	ND	ND	ND	ND	1 (1.7%)
Dónofrio et al.	2016	SuAVR Perceval	214	ND	ND	ND	2	0.6	11.5	2.3	ND	ND	211 (98.6%)	ND	5 (2.3%)
		TAVI	214	ND	ND	ND	1.5	0.5	7	1.17	ND	ND	190 (88.8%)	ND	8 (3.7%)
Miceli et al.	2016	SuAVR Perceval	37	0 (0%)	0 (0%)	ND	1.25	0.38	7	0.6	ND	ND	ND	0 (0%)	ND
		TAVI	37	2 (5.4%)	1 (2.7%)	ND	1	0.2	4.5	0.88	ND	ND	ND	3 (8.1%)	ND
Muneretto et al.	2020	SuAVR Perceval	291	ND	0 (0%)	0 (0%)	ND	ND	ND	ND	ND	ND	ND	ND	4 (1.4%)
		TAVI	291	ND	1 (0.3%)	23 (7.9%)	ND	ND	ND	ND	ND	ND	ND	ND	16 (5.5%)
Biancari et al.	2016	SuAVR Perceval	144	0 (0%)	ND	0 (0%)	ND	ND	ND	ND	ND	ND	115 (79.9%)	2 (1.4%)	ND
		TAVI	144	0 (0%)	ND	15 (10.4%)	ND	ND	ND	ND	ND	ND	112 (77.8%)	10 (6.9%)	ND
Zubarevich et al.	2022	SuAVR Perceval	56	ND	ND	ND	2.1	0.8	9.7	5.3	ND	ND	ND	ND	1 (1.8%)
		TAVI	56	ND	ND	ND	2.5	0.5	8.2	3.4	ND	ND	ND	ND	0 (0%)
Santarpino et al.	2022	SuAVR Perceval	172	0 (0%)	ND	ND	ND	ND	12	8	ND	ND	ND	7 (4%)	ND
		TAVI	172	0 (0%)	ND	ND	ND	ND	8	5	ND	ND	ND	5 (2.9%)	ND
Santarpino et al.	2015	SuAVR Perceval	102	1 (1%)	1 (!%)	0 (0%)	2.2	2.7	12	6	ND	ND	ND	5 (4.9%)	ND
		TAVI	102	2 (2%)	0 (0%)	5 (4.9%)	3.2	3.5	14	6	ND	ND	ND	3 (2.9%)	ND
Indelen et al.	2023	SuAVR Perceval	61	ND	ND	0 (0%)	ND	ND	ND	ND	ND	ND	ND	1 (1.6%)	ND
		TAVI	53	ND	ND	1 (1.8%)	ND	ND	ND	ND	ND	ND	ND	2 (3.8%)	ND
Muneretto et al.	2023	SuAVR Perceval	517	ND	ND	4 (0.8%)	0.87	0.47	ND	ND	1.55	0.03	ND	ND	11 (2.1%)
		TAVI	517	ND	ND	29 (5.6%)	1	0.6	ND	ND	1.52	0.04	ND	ND	24 (4.6%)

Table S7. Reported clinical outcomes and complications after SUAVR or TAVI of included articles for the first part of the study (Continue 2).

First Author	Intervention Groups	Sample Size	Duration	Cardiovascular Mortality	All-Mortality	Stroke	HF Hospitalization	MI	PPI	Endokarditis	Prothesis-Failure and need to Re-OP/Explant
Vilalta et al.	SuAVR Perceval	171	2-Year	ND	31 (18.1%)	11 (6.4%)	31 (18.1%)	2 (1.2%)	23 (13.5%)	ND	ND
	TAVI	171	2-Year	ND	17 (9.9%)	8 (4.7%)	4 (2.3%)	5 (2.9%)	34 (20%)	ND	ND
Chung et al.	SuAVR Perceval	62	1-Year	1 (1.7%)	1 (1.7%)	5 (8.3%)	2 (3.3%)	0 (0%)	7 (11.7%)	1 (1.8%)	ND
	TAVI	62	1-Year	1 (1.7%)	4 (7%)	2 (3.2%)	3 (5.1%)	0 (0%)	9 (14.5%)	0 (0%)	ND
Miceli et al.	SuAVR Perceval	37	2-Year	survival report		ND	ND	ND	ND	ND	ND
	TAVI	37	2-Year			ND	ND	ND	ND	ND	ND
Zubarevich et al.	SuAVR Perceval	56	1-Year	ND	3 (5.3%)	ND	ND	ND	ND	ND	ND
	TAVI	56	1-Year	ND	1 (1.7%)	ND	ND	ND	ND	ND	ND
Santarpino et al.	SuAVR Perceval	172	5-Year	survival report		ND	ND	ND	ND	ND	ND
	TAVI	172	5-Year			ND	ND	ND	ND	ND	ND
Santarpino et al.	SuAVR Perceval	102	5-Year	survival report		ND	ND	ND	ND	ND	ND
	TAVI	102	5-Year			ND	ND	ND	ND	ND	ND
Dónofrio et al.	SuAVR Perceval	214	1-Year	survival report							
	TAVI	214	1-Year								
Indelen et al.	SuAVR Perceval	61	1-Year	survival report		ND	ND	ND	ND	ND	ND
	TAVI	53	1-Year			ND	ND	ND	ND	ND	ND
Muneretto et al.	SuAVR Perceval	517	5-Year	survival report until 9 years		ND	ND	ND	ND	ND	ND
	TAVI	517	5-Year			ND	ND	ND	ND	ND	ND

Table S8. Demographic Data of included articles for the second part of the study.

First Name	Year	County	Center	Study Period	Name of Trial	Sample Size (Patient with Perceval)	Median-Follow-UP (Year)	Gender Male (Nr)	NYHA I (Nr)	NYHA II (Nr)	NYHA III (Nr)	NYHA IV (Nr)	EuroScore	STS-Score	HTN (Nr)	Choltesrol/HLP (Nr)	Smoker (Nr)	[illegible]
Aldea et al.	2023	USA	Multicenter	2010-2015	Retrospective observational study	970	4 years	415	ND	ND	545		ND	5.7±6.5	806	669	ND	[illegible]
Concistre et al.	2023	Italy	Single-Center	2011-2021	Prospective cohort study	1652	1 year (up to 8 years)	761	ND	1361		76	4.1±6.3	ND	ND	937	ND	[illegible]
Dokollari et al.	2023	Netherlands	Single-Center	2013-2020	Retrospective observational study	101	7 years	55	ND	ND	ND	ND	3.51±4.48	ND	80	76	40	[illegible]
Ferreira et al.	2022	Portugal	Single-Center	2015-2020	Retrospective observational study	196	Up to 5 years	109	ND	ND	ND	ND	2.91±2.20	ND	173	140	37	[illegible]
Fischlein et al.	2021	Germany	Multicenter	2010-2013	Prospective cohort study	658	3.8 years	234	22	198	386	32	10.2±7.8	7.2±7.4	551	ND	31	[illegible]
Glauber et al.	2020	Italy	Multicenter	2011-2018	Prospective cohort study	480	2.4 years	171	19	284	158	13	7.87±1.7	MD	ND	291	ND	[illegible]
Hong	2024	Korea	Single-Center	2015-2020	Retrospective observational study	113	51.19±20.6	48	14	58	33	8	ND	10.4±3.9	81	47	ND	[illegible]
Kapadia	2024	UK	Single-Center	2014-2020	Retrospective observational study	102	ND	62	ND	ND	ND	ND	2.1±0.4	ND	74	ND	57	[illegible]
Lamberigts et al.	2022	Belgium	Single-Center	2007-2019	Retrospective observational study	784	7.03 years	279	45	333	342	64	4.55±1.35	ND	ND	ND	ND	[illegible]
Meuris et al.	2015	Belgium	Multicenter	2007-2008	Prospective cohort study	30	4.2 years	8	0	0	28	2	13.18±7.28	ND	ND	ND	ND	[illegible]
Muneretto et al.	2022	Italy	Multicenter	2008-2015	Retrospective observational study	481	5 years	174	ND	ND	285		13.7±1.54	5.7±1.17	394	ND	ND	[illegible]
Pollari et al.	2023	Germany	Single-Center	2010-2020	Retrospective observational study	547	4.53 years	268	ND	ND	ND	ND	3.4±2.6		493	409	112	[illegible]

Santarpino et al.	2022	Italy	Multicenter	2010-2018	Retrospective observational study	172	6.1 years	146	ND	ND	ND	ND	3.4±2.3	ND	99	ND	ND	[illegible]
Schizas et al.	2024	Greece	Single-Center	2013-2020	Retrospective observational study	205	6.27±2.03	70	ND	ND	ND	ND	5.85±3.85	ND	ND	ND	ND	[illegible]
Szecel et al.	2021	Belgium	Single-Center	2007-2017	Retrospective observational study	468	3.1±2 up to 11.2 year	206	26	164	231	47	5.1±5.5	5.8±5.5	ND	ND	ND	[illegible]
White et al.	2022	Canada	Single-Center	2013-2019	Retrospective observational study	295	2.4 years	188	ND	ND	ND	ND	ND	ND	188	180	14	[illegible]

Table S9. Information about surgical related demographics for the second part of the study.

First Name	Year	Tricuspid aortic valve (Nr)	Bicuspid aortic valve (Nr)	Full-Sternotomy (Nr)	Minimally Invasive (Nr)	Minithorakotomy (Nr)	Ministernotomy (Nr)	Re-Sternotomy (Nr)	Prior CABG (Nr)	Prior Valve-OP (Nr)	Aortic Stenosis (Nr)	Aortic Regurgit (Nr)
Aldea et al.	2023	934	36	669	301	ND	ND	35	26	9	611	3
Concistre et al.	2023	1520	132	899	744	324	420	24	270 orevious cardiac procedures		1233	89
Dokollari et al.	2023	76	25	101	0	0	0	24	ND	ND	24	76
Ferreira et al.	2022	ND	ND	181	15	15	0	4	ND	ND	189	3
Fischlein et al.	2021	658	12	439	219	3	216	34	13	11	430	2
Glauber et al.	2020	433	47	0	480	266	214	ND	5	15	359	11
Hong	2024	100	13	39	27	2	25	ND	2	10	86	ND
Kapadia	2024	ND	ND	74	28	ND	28	ND	ND	ND	89	ND
Lamberigts et al.	2022	ND	ND	541	243	16	227	ND	ND	ND	ND	ND
Meuris et al.	2015	ND	ND	30	0	0	0	3	previous cardiac surgery 3		23	ND
Muneretto et al.	2022	450	31	256	225	94	131	35	18	ND	481	0
Pollari et al.	2023	ND	69	162	385	9	376	21	18	3	ND	ND
Santarpino et al.	2022	ND	ND	ND	ND	ND	ND	ND	ND	ND	172	0
Schizas et al.	2024	205	ND	28	109	0	109	ND	ND	ND	185	4
Szecel et al.	2021	457	11	328	140	14	126	ND	ND	ND	ND	8
White et al.	2022	ND	ND	ND	ND	ND	ND	ND	10	ND	ND	ND

Table S10. Information about SUAVR for the second part of the study.

First Name	Year	Isolated AVR (Nr)	Concomitant AVR (Nr)	Overall ACC min	isolated ACC min	concomitant ACC min	Overal CPB min	isolated CBP min	concomitant CBP min	S	M	L
Aldea et al.	2023	646	324	ND	38.5±13.5	57.8±23.5	ND	60.8±20.9	83.2±30	101	387	387
Concistre et al.	2023	1059	593	61±29.9	51±20.5	79.1±35.2	90.3±42.2	77.4±30.8	113.3±49.4	241	537	594
Dokollari et al.	2023	101	0	47.31±21.3	47.31±21.3	ND	65±29.6	65±29.6	ND	23	35	31
Ferreira et al.	2022	122	74	33.31±14.09	ND	ND	45.55±19.04	ND	ND	28	69	64
Fischlein et al.	2021	418	210	40.7±18.1	35.5±12.4	52.3±22.9	64.8±25.2	58.7±20.2	78.7±29.4	84	290	255
Glauber et al.	2020	457	23	51±17	ND	ND	81±36.7	ND	ND	72	151	203
Hong	2024	66	40	ND	35.2±3.9	77.6±31.7	ND	82.7±9.5	119.1±40.5	15	44	37
Kapadia	2024	54	48	59.7±2.89	52.5±5.7	75.25±8.3	75.25±8.3	81.75±8.36	108.25±15.29	17	39	34
Lamberigts et al.	2022	349	435	55.75±13.5	38.25±3.76	69.5±12.12	85.5±16.7	61.45±6.29	105.75±14.13	63	267	291
Meuris et al.	2015	16	14	ND	29.3±8	45.4±15.4	ND	46.4±6.7	73.3±21.8	11	19	ND
Muneretto et al.	2022	481	0	35±16	35±16	ND	56±25	56±25	ND	ND	ND	ND
Pollari et al.	2023	374	173	ND	36.1±11	53.4±22	ND	59.4±20	83.6±28	46	185	219
Santarpino et al.	2022	172	0	ND	59.56±29.2	ND	ND	80.63±45.5	ND	ND	ND	ND
Schizas et al.	2024	137	68	68.2±61.5	49.1±13.4	ND	108.3±63.5	59.1±15.3	ND	29	84	59
Szecel et al.	2021	201	267	61±30	39±19	79±32	94±40	66±22	118±40	29	159	173
White et al.	2022	201	90	73-8±37.5	ND	ND	108.3±56.4	ND	ND	ND	ND	ND

Table S11. Echocardiographic Data of included articles for the second part of the study.

First Name	Year	1-Year		2-Year		3-Year		4-Year		5-Year	
		**MVG**	**PVG**	**MVG**	**PVG**	**MVG**	**PVG**	**MVG**	**PVG**	**MVG**	**PVG**
Aldea et al.	2023	6.8±5	12.6±9.4	8±5	14.6±9	9.4±5.8	17.3±9.8	9.6±5.7	18.5±10.3	9.4±6.6	18.5±11.8
Concistre et al.	2023	11.9±5.5	20.5±8.9	ND	ND	ND	ND	ND	ND	13.7±10	23±15
Dokollari et al.	2023	ND	ND	ND	ND	ND	ND	ND	ND	ND	ND
Ferreira et al.	2022	11.88±4.39	ND	ND	ND	ND	ND	ND	ND	ND	ND
Fischlein et al.	2021	9.1±5	17.1±8.7	9.3±5	17.1±8.4	9.3±5.8	17.2±9.8	9.6±5.7	18.5±10.4	9±6.3	17.8±11.3
Glauber et al.	2020	11.6±5.1	ND	11.3±5.4	ND	11.3±5.4	ND	12.6±6.2	ND	13.6±8.6	ND
Hong	2024	ND	ND	ND	ND	ND	ND	ND	ND	ND	ND
Kapadia	2024	ND	ND	ND	ND	ND	ND	ND	ND	ND	ND
Lamberigts et al.	2022	ND	ND	ND	ND	ND	ND	ND	ND	ND	ND
Meuris et al.	2015	9.9±4.6	20.9±9.2	8±4.1	16.6±7.2	8.3±2.5	16.6±6.2	7.6±3.6	17.5±7.8	9.3±5.5	21.4±11.5
Muneretto et al.	2022	10.9±5.9	21.26±9.5	11.2±6.1	21.5±9.2	11.6±5.8	21.9±9.1	12±5.4	22.1±9.1	12.2±5.7	22.3±9
Pollari et al.	2023	12±4	22±9	ND	ND	ND	ND	ND	ND	ND	ND
Santarpino et al.	2022	ND	ND	ND	ND	ND	ND	ND	ND	ND	ND
Schizas et al.	2024	ND	ND	ND	ND	ND	ND	ND	ND	ND	ND
Szecel et al.	2021	ND	ND	ND	ND	ND	ND	ND	ND	ND	ND
White et al.	2022	ND	ND	ND	ND	ND	ND	ND	ND	ND	ND

Supplementary Materials. Figures

Figure S1. Subgroup analysis based on minimally invasive SUAVR

A

Study or Subgroup	Perceval SuAVR Mean	Perceval SuAVR SD	Perceval SuAVR Total	TAVI Mean	TAVI SD	TAVI Total	Weight	Mean Difference IV, Random, 95% CI	Year
Santarpino et al. 2015	12	6	102	14	6	102	31.5%	−2.00 [−3.65, −0.35]	2015
D'Onofrio et al. 2016	11.5	2.3	214	7	1.17	214	0.0%	4.50 [4.15, 4.85]	2016
Miceli et al. 2016	7	0.6	37	4.5	0.88	37	35.8%	2.50 [2.16, 2.84]	2016
Chung et al. 2021	13.1	8.8	62	7.1	7.9	62	0.0%	6.00 [3.06, 8.94]	2021
Gerfer et al. 2021	12	5	59	9	5	59	0.0%	3.00 [1.20, 4.80]	2021
Zubarevich et al. 2022	9.7	5.3	56	8.2	3.4	56	0.0%	1.50 [−0.15, 3.15]	2022
Santarpino et al. 2022	12	8	172	8	5	172	32.6%	4.00 [2.59, 5.41]	2022
**Total (95% CI)**			**311**			**311**	**100.0%**	**1.57 [−1.04, 4.18]**	

Heterogeneity: $Tau^2 = 4.93$; $Chi^2 = 32.70$, df = 2 ($P < 0.00001$); $I^2 = 94\%$
Test for overall effect: Z = 1.18 (P = 0.24)

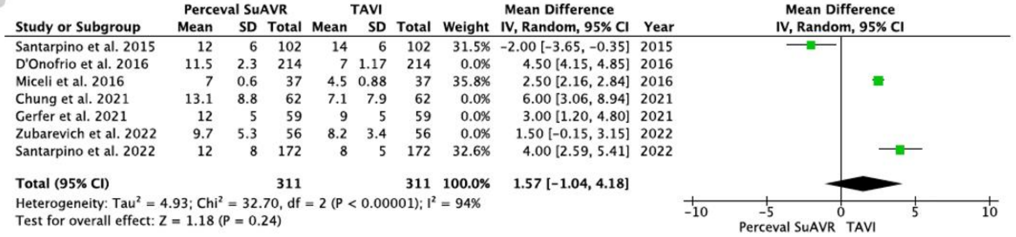

B

Study or Subgroup	Perceval SuAVR Mean	Perceval SuAVR SD	Perceval SuAVR Total	TAVI Mean	TAVI SD	TAVI Total	Weight	Mean Difference IV, Random, 95% CI	Year
D'Onofrio et al. 2016	13.7	6.6	214	11	4.6	214	0.0%	2.70 [1.62, 3.78]	2016
Miceli et al. 2016	11.4	3.7	37	10.1	3.4	37	38.3%	1.30 [−0.32, 2.92]	2016
Vilalta et al. 2021	16.8	6.9	171	11.4	5.7	171	0.0%	5.40 [4.06, 6.74]	2021
Chung et al. 2021	14.7	3.8	62	11.2	5.7	62	0.0%	3.50 [1.79, 5.21]	2021
Gerfer et al. 2021	12	4	59	9	4	59	0.0%	3.00 [1.56, 4.44]	2021
Zubarevich et al. 2022	5.8	0.8	56	5	0.6	56	0.0%	0.80 [0.54, 1.06]	2022
Muneretto et al. 2023	10.6	6.2	517	10.8	6.5	517	61.7%	−0.20 [−0.97, 0.57]	2023
**Total (95% CI)**			**554**			**554**	**100.0%**	**0.37 [−1.05, 1.80]**	

Heterogeneity: $Tau^2 = 0.71$; $Chi^2 = 2.68$, df = 1 ($P = 0.10$); $I^2 = 63\%$
Test for overall effect: Z = 0.51 (P = 0.61)

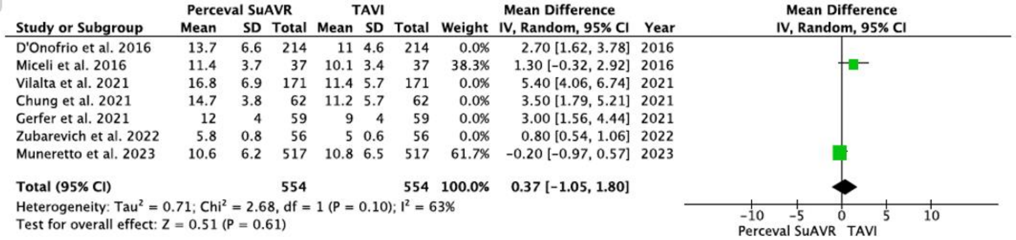

Figure S2. Comparison of mid-term outcomes between SUAVR and TAVI

A

Study or Subgroup	Perceval SuAVR Events	Perceval SuAVR Total	TAVI Events	TAVI Total	Weight	Odds Ratio M-H, Random, 95% CI	Year
Vilalta et al. 2021	23	171	34	171	77.0%	0.63 [0.35, 1.12]	2021
Chung et al. 2021	7	61	9	62	23.0%	0.76 [0.27, 2.20]	2021
**Total (95% CI)**		**232**		**233**	**100.0%**	**0.66 [0.39, 1.09]**	
Total events	30		43				

Heterogeneity: $Tau^2 = 0.00$; $Chi^2 = 0.10$, df = 1 (P = 0.75); $I^2 = 0\%$
Test for overall effect: Z = 1.63 (P = 0.10)

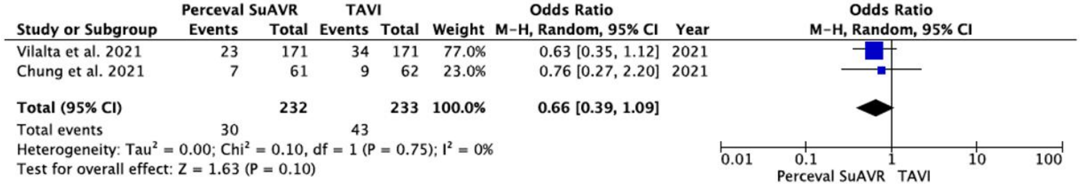

B

Study or Subgroup	Perceval SuAVR Events	Perceval SuAVR Total	TAVI Events	TAVI Total	Weight	Odds Ratio M-H, Random, 95% CI	Year
Vilalta et al. 2021	11	171	8	171	76.3%	1.40 [0.55, 3.57]	2021
Chung et al. 2021	5	62	2	62	23.7%	2.63 [0.49, 14.11]	2021
**Total (95% CI)**		**233**		**233**	**100.0%**	**1.63 [0.72, 3.69]**	
Total events	16		10				

Heterogeneity: $Tau^2 = 0.00$; $Chi^2 = 0.41$, df = 1 (P = 0.52); $I^2 = 0\%$
Test for overall effect: Z = 1.17 (P = 0.24)

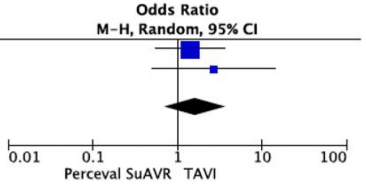

C

Study or Subgroup	Perceval SuAVR Events	Perceval SuAVR Total	TAVI Events	TAVI Total	Weight	Odds Ratio M-H, Random, 95% CI	Year
Santarpino et al. 2015	96	97	92	99	6.2%	7.30 [0.88, 60.53]	2015
Miceli et al. 2016	34	37	24	37	10.0%	6.14 [1.58, 23.91]	2016
D'Onofrio et al. 2016	202	214	194	214	14.4%	1.74 [0.83, 3.65]	2016
Vilalta et al. 2021	140	171	154	171	15.1%	0.50 [0.26, 0.94]	2021
Chung et al. 2021	61	62	58	62	5.9%	4.21 [0.46, 38.76]	2021
Zubarevich et al. 2022	53	56	55	56	5.6%	0.32 [0.03, 3.19]	2022
Santarpino et al. 2022	150	172	98	172	15.7%	5.15 [3.00, 8.83]	2022
Indelen et al. 2023	57	61	48	53	10.0%	1.48 [0.38, 5.84]	2023
Muneretto et al. 2023	390	517	367	517	17.1%	1.26 [0.95, 1.65]	2023
**Total (95% CI)**		**1387**		**1381**	**100.0%**	**1.89 [0.98, 3.65]**	
Total events	1183		1090				

Heterogeneity: $Tau^2 = 0.64$; $Chi^2 = 42.28$, df = 8 (P < 0.00001); $I^2 = 81\%$
Test for overall effect: Z = 1.90 (P = 0.06)

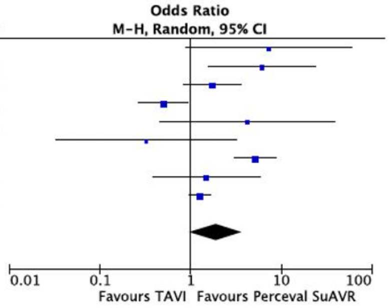

Figure S3. Explantation of Perceval Bioprosthesis at mid-term follow-up

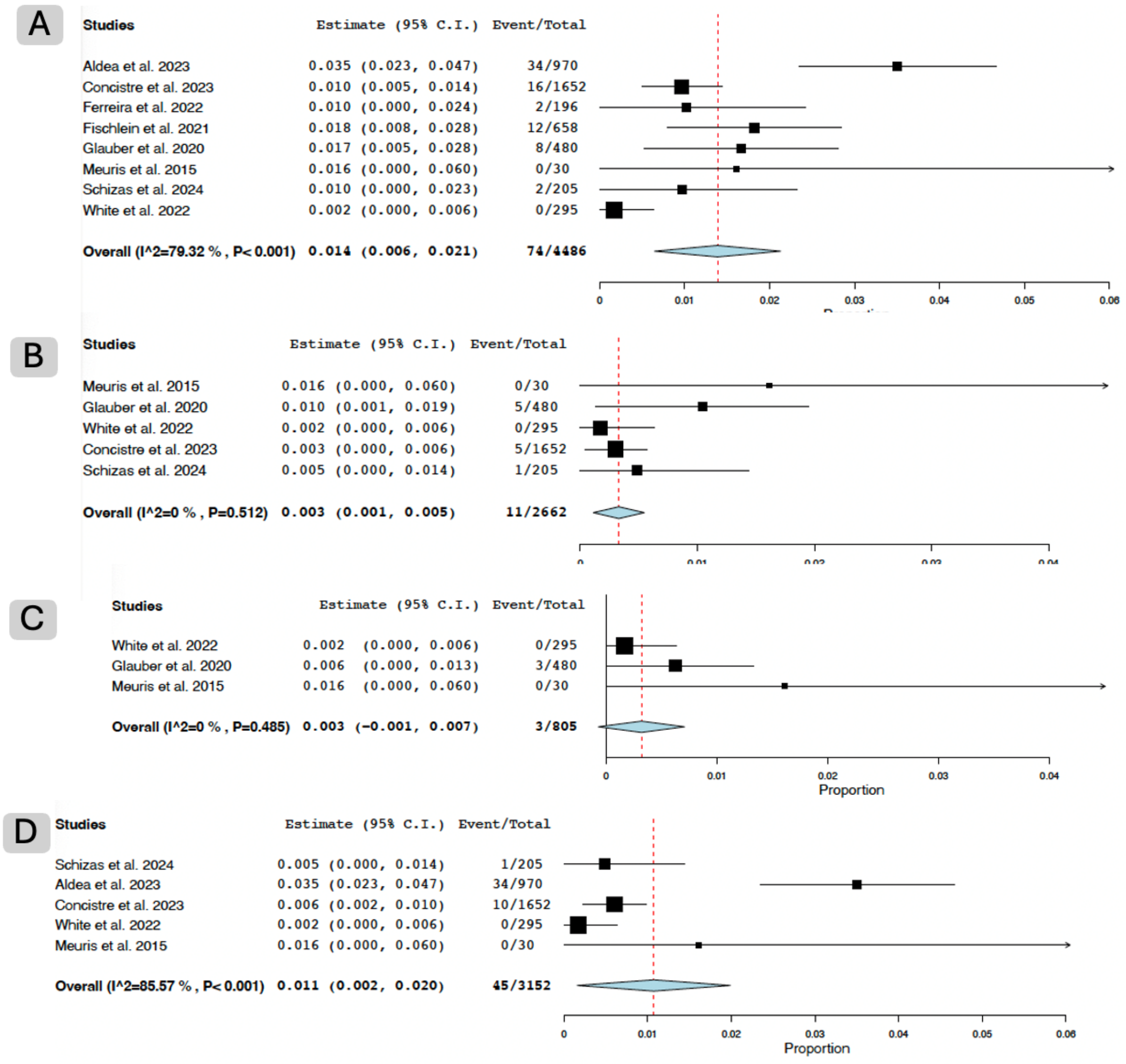

Figure S4. Long-term Outcomes Following SUAVR

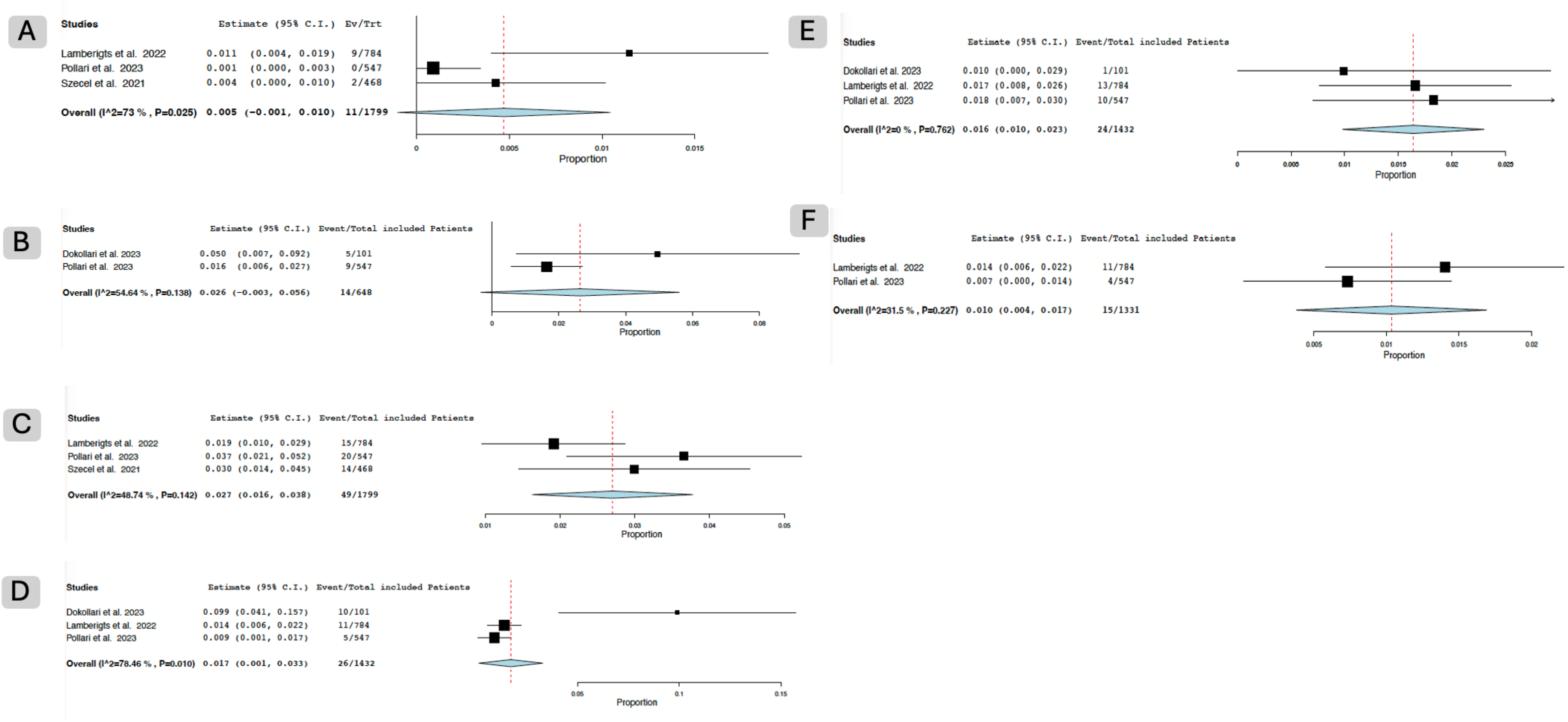

Figure S5. Life expectancy after SUAVR

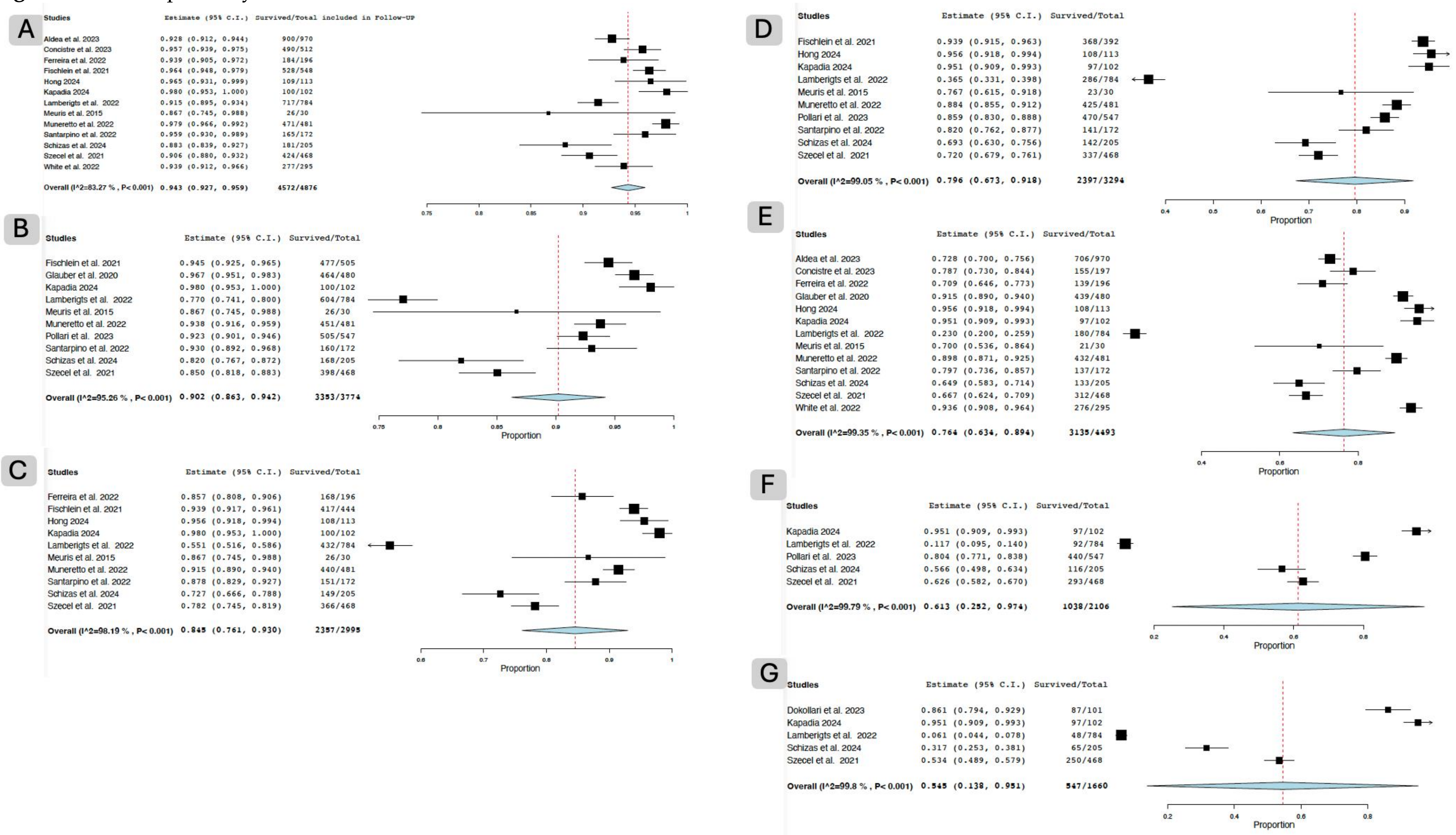

Supplement: Supplementary file 1 [file jcm-13-04887-s001.zip › jcm-3159210-supplementary.pdf]
